# Sexual Preferences in Nutrient Utilization Regulate Oxygen Consumption and Reactive Oxygen Species Generation in *Schistosoma mansoni*: Potential Implications for Parasite Redox Biology

**DOI:** 10.1371/journal.pone.0158429

**Published:** 2016-07-05

**Authors:** Matheus P. Oliveira, Juliana B. R. Correa Soares, Marcus F. Oliveira

**Affiliations:** Laboratório de Bioquímica de Resposta ao Estresse, Instituto de Bioquímica Médica Leopoldo de Meis, Universidade Federal do Rio de Janeiro, Cidade Universitária, Rio de Janeiro, Brazil; University of California Los Angeles, UNITED STATES

## Abstract

*Schistosoma mansoni*, one of the causative agents of human schistosomiasis, has a unique antioxidant network that is key to parasite survival and a valuable chemotherapeutic target. The ability to detoxify and tolerate reactive oxygen species increases along *S*. *mansoni* development in the vertebrate host, suggesting that adult parasites are more exposed to redox challenges than young stages. Indeed, adult parasites are exposed to multiple redox insults generated from blood digestion, activated immune cells, and, potentially, from their own parasitic aerobic metabolism. However, it remains unknown how reactive oxygen species are produced by *S*. *mansoni* metabolism, as well as their biological effects on adult worms. Here, we assessed the contribution of nutrients and parasite gender to oxygen utilization pathways, and reactive oxygen species generation in whole unpaired adult *S*. *mansoni* worms. We also determined the susceptibilities of both parasite sexes to a pro-oxidant challenge. We observed that glutamine and serum importantly contribute to both respiratory and non-respiratory oxygen utilization in adult worms, but with different proportions among parasite sexes. Analyses of oxygen utilization pathways revealed that respiratory rates were high in male worms, which contrast with high non-respiratory rates in females, regardless nutritional sources. Interestingly, mitochondrial complex I-III activity was higher than complex IV specifically in females. We also observed sexual preferences in substrate utilization to sustain hydrogen peroxide production towards glucose in females, and glutamine in male worms. Despite strikingly high oxidant levels and hydrogen peroxide production rates, female worms were more resistant to a pro-oxidant challenge than male parasites. The data presented here indicate that sexual preferences in nutrient metabolism in adult *S*. *mansoni* worms regulate oxygen utilization and reactive oxygen species production, which may differently contribute to redox biology among parasite sexes.

## Introduction

The trematode *Schistosoma mansoni* is a long-living intravascular parasite and a major causative agent of human schistosomiasis, a chronic disease afflicting more than 240 million people worldwide [1]. Clinically, this illness is manifested in two distinct phases: an early acute one, when infected individuals exhibit mild symptoms such as fever, diarrhea and cough [2], and a late chronic phase, when severe clinical features including liver fibrosis, portal hypertension and hepatosplenomegaly, manifest [3]. These events are consequence of an intense inflammatory granulomatous reaction elicited by egg antigens deposited in intestine and liver [4–6]. Indeed, adult *S*. *mansoni* worms living in the mesenteric blood vessels are directly targeted by the host immune response [7,8]. Despite this, adult worms can live for decades within humans, implying the existence of efficient evasive mechanisms to counteract host immune response, which include *i)* reduced expression of antigenic proteins and the sequestering of host erythrocyte glycolipids at tegument surface [9,10], *ii)* the ability to mediate complement C3 degradation [8], *iii)* and the build-up of parasite antioxidant defenses along development [11–14].

Reactive oxygen species (ROS) are naturally produced in distinct cell compartments, such as in peroxisomes [15], in mitochondria [16], and at plasma membrane [17], playing key signaling roles in determining cell fate, growth and survival [18,19]. ROS have also been directly implicated as part of the immune arsenal in sterile inflammation, and during infections, mediating pathogen killing [20]. Interestingly, the observed increase in hydrogen peroxide (H_2_O_2_) detoxification capacity and resistance to multiple sources of ROS along *S*. *mansoni* development in the vertebrate host results from improved parasite antioxidant defenses [11–14]. This suggests that adult parasites are more exposed to redox challenges than young stages [21,22], which might include pro-oxidant products derived from physiological blood digestion [23–26], and ROS generated by activated immune cells [21,22] and, potentially, from parasite aerobic energy metabolism. In fact, adult *S*. *mansoni* are exposed to ROS produced by circulating immune cells, which play an essential role in parasite killing [21,22,27]. Thus, compelling evidence indicate that increased ability to detoxify ROS in *S*. *mansoni* represent an adaptive mechanism to avoid redox imbalance and parasite cell death triggered by host immune system.

Despite a clear relationship between energy and redox metabolism was established over the years for many organisms [16,28–35], knowledge on this metabolic intersection, and the potential consequences to basic aspects of trematode parasite biology were scarcely investigated in the literature [36–42]. Indeed, adult *S*. *mansoni* worms, living in the vertebrate bloodstream, are continuously exposed to molecular oxygen (O_2_), which is known to be physiologically utilized by the parasites to sustain egg production [43,44] and energy demand through the oxidative phosphorylation (OXPHOS) [42,45,46–55]. Regarding the nutrient requirements, it is known that carbohydrates [55], and particularly glutamine metabolism, play a key role to maintain parasite energy homeostasis, motility, tegumental resting membrane potential, muscle tension and viability in adult worms [56–59]. Although adult *S*. *mansoni* cannot synthesize *de novo* long chain fatty acids, they are able to produce complex lipids from host lipid sources [60,61]. Recently, fatty acid oxidation was demonstrated to play a central energetic role in adult female worms to support egg production, but not their viability, since genetic or pharmacological interference on lipolysis/β-oxidation drastically reduced parasite respiration and oogenesis [42,62].

Schistosomiasis control and treatment programs have relied for decades on a single therapeutic agent (praziquantel) [63]. However, growing concerns on the potential development of resistance to this drug [64] have prompted research on novel targets against *S*. *mansoni* [25,65–70]. Results from these efforts revealed the complexity, importance and uniqueness of antioxidant defense mechanism to parasite survival [65–72]. Noteworthy, beyond the presence of classical antioxidant enzymes [11,13,72,73] and the complete lack of catalase, *S*. *mansoni* also express specific 2-cys peroxiredoxins (Prx) 2 and 3, which catalyses the decomposition of H_2_O_2_ and organic hydroperoxides using both reduced glutathione (GSH) and thioredoxin (Trx) as electron donors [67,71,73]. Also, a unique thioredoxin glutathione reductase (TGR), which inter-convert reduced and oxidized forms of both GSH and Trx, plays a key antioxidant role [66,68–70]. Indeed, a number of strategies targeting specifically *S*. *mansoni* redox metabolism were designed and validated as new tools to treat schistosomiasis [25,65,68–70,74,75]. However, despite the knowledge on *Schistosoma* redox biology, the physiological sources of ROS, and their biological effects to adult *S*. *mansoni* worms remain largely unknown.

*Schistosoma* parasites are dioecious, with two genetically defined and dimorphic sexes [76–78], and these differences can be extended to worm energy and redox metabolism [47,52,57]. For example, glutamate, aspartate, and alanine uptake were reportedly higher in males [52,57], while no apparent gender trend was observed in glucose uptake [47,52,79–81]. Also, the rates of glucose, glutamate, and alanine oxidation were higher in males of both *S*. *mansoni* and *S*. *japonicum*, compared to females [52,57,82], suggesting increased OXPHOS in males. Beyond these mechanisms, two interesting metabolic features were also observed in adult worms: *i)* that copula regulates glucose metabolism [82–84] in a way that paired parasites exhibit higher glucose uptake than separated ones [83]; *ii)* males were able to transfer glycogen-derived glucose to females [83,84]. Regarding redox metabolism, evidence indicate that females contain higher levels of antioxidant mechanisms than males [67,78,85–87], including higher expression of ferredoxin NADP(H) oxidoreductase [85], peroxiredoxins 1,2, and 3 [67], Trx [78,86], TGR [78], and superoxide dismutases [78,87]. Indeed, the trend is that female worms are more dependent on antioxidant defenses than males, since depletion of GSH by oltipraz resulted in lower worm burden, particularly females [65], while simultaneous treatment with H_2_O_2_ and distinct antioxidant enzyme inhibitors more intensely affected female than male worms [12].

Despite redox metabolism having been validated as a key chemotherapeutic target against schistosomiasis, and that parasites use distinct nutrients and O_2_ to meet their energy requirements [42–59], it remains elusive how endogenous ROS are produced by the parasite’s own metabolism and their potential biological consequences. Here, we investigated how nutrients and gender regulate O_2_ utilization by distinct pathways and ROS generation in whole unpaired adult *S*. *mansoni* worms. We also assessed the susceptibilities to a pro-oxidant challenge among parasite sexes. The data shown here indicate that unpaired adult *S*. *mansoni* worms exhibit sexual preferences in substrate utilization that modulate O_2_ consumption and ROS generation pathways which might provide differential tolerance to oxidative stress among parasite sexes.

## Methods

### Ethics statement

All animal experiments were overseen and approved by the Commission for Evaluation of Animal Use for Research from the Federal University of Rio de Janeiro (CAUAP-UFRJ, protocol number IBQM#050), under strict accordance to the Guide for the Care and Use of Laboratory Animals [88]. Female Swiss mice at four weeks of age were obtained from Fundawer Instituto Oswaldo Cruz breeding colony (Fiocruz-RJ, Brazil) and housed at Institute of Medical Biochemistry Leopoldo de Meis (IBqMLdM-UFRJ) animal care facility. Mice were kept in a 12/12-hr light/dark cycle, fed with regular chow diet and water *ad libitum* and care was taken to minimize pain and suffering of the animals. Technicians dedicated to the IBqMLdM-UFRJ animal care facility carried out all aspects related to mice husbandry under strict guidelines to ensure careful and consistent handling of the animals.

### Parasites

*S*. *mansoni* cercariae (LE strain) were obtained from infected *Biomphalaria glabrata* snails at Department of Malacology (Fiocruz-RJ, Brazil), as previously described [89]. Mice were infected by subcutaneous injection of 1 mL of water containing about 150 cercariae using an insulin syringe and then kept at IBqMLdM-UFRJ animal care facility for about forty-two days. Then, mice were humanely euthanized by CO_2_ asphyxiation, and all efforts were made to minimize animal suffering. Adult worm pairs were manually collected from mice mesenteries and then pre-incubated for 1h at 37°C and 5% CO_2_ in one of the following "pre-incubation media": *i*) Hank’s Balanced Salt Solution (HBSS) containing 5.5 mM Glucose (hereafter named Glc); *ii*) HBSS containing 5.5 mM Glucose and 5.5 mM Glutamine (hereafter named Glc+Gln); *iii*) RPMI 1640 (LCG Biotecnologia, Brazil) containing 11 mM Glucose, 2.05 mM Glutamine, several aminoacids and vitamins (hereafter named RPMI); *iv*) RPMI containing 11 mM Glucose, 2.05 mM Glutamine, several aminoacids, vitamins and supplemented with 10% fetal bovine serum (hereafter named RPMI+Serum). During pre-incubation, worm pairs spontaneously separated from each other and sex-specific sets of worms (male or female) were utilized for all the subsequent experiments.

### Respirometry analyses

**Fig 1:**
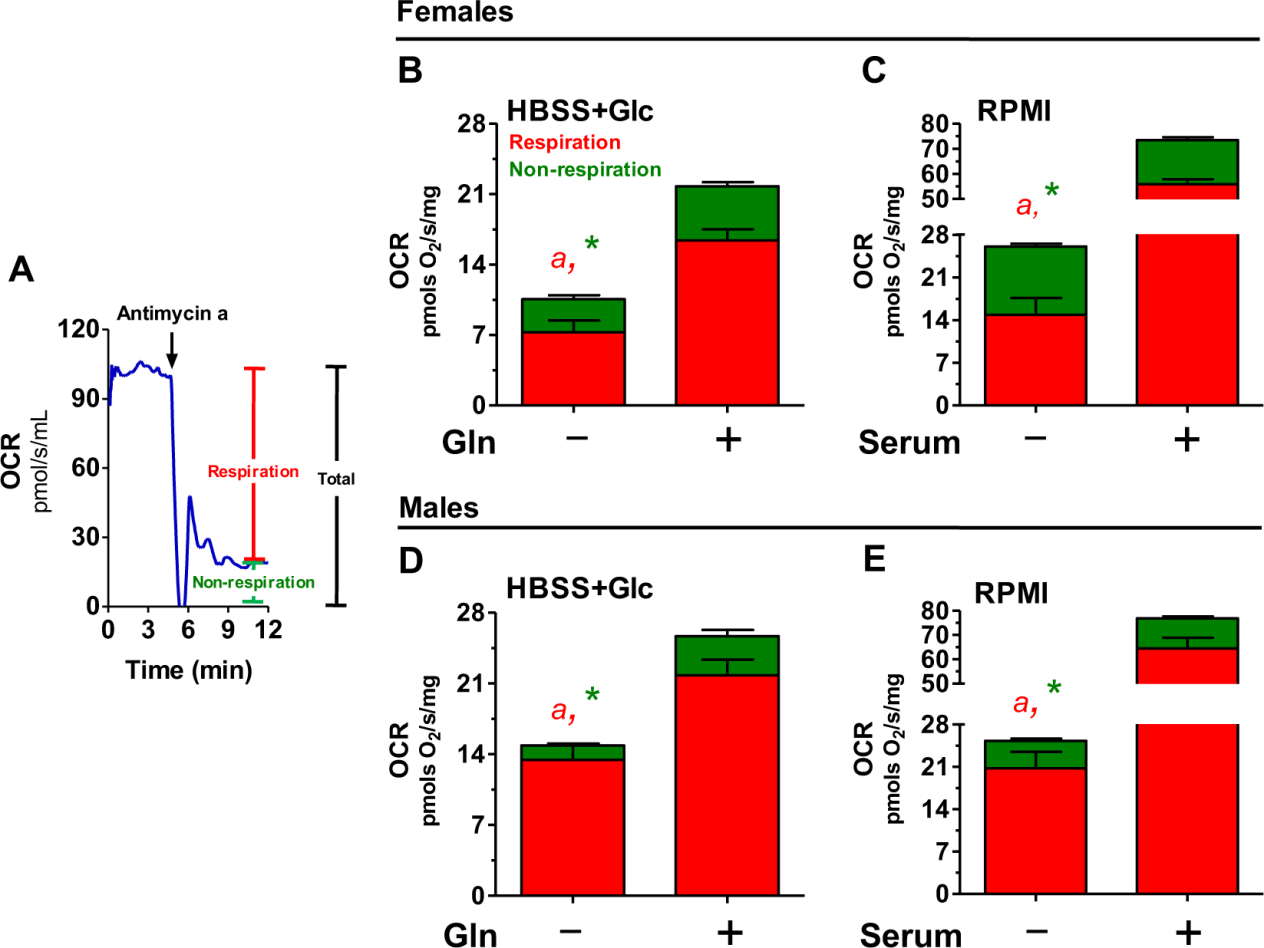
Nutrients regulate respiratory and non-respiratory pathways in adult *S*. *mansoni* worms: O_2_ consumption rates (OCR) of intact adult *S*. *mansoni* worms were determined by high resolution respirometry in media containing four different nutrient compositions as following: HBSS containing 5.5 mM glucose (Glc), HBSS containing 5.5 mM glucose plus 5.5 mM glutamine (+Gln), RPMI 1640 (RPMI), RPMI 1640 containing 10% fetal bovine serum (+Serum). (A) Representative OCR trace obtained from 30 intact *S*. *mansoni* males in RPMI 1640. To determine the contribution of different pathways of O_2_ utilization, total OCR was measured followed by addition of 6 μg/mL antimycin a (AA) to the respirometer chamber. The AA-sensitive component of the OCR was considered as "respiration", while the AA-insensitive OCR, as "non-respiration". In Fig B, C, D and E red bars represent respiration while green bars, non-respiration OCR determined in four media composition. Data are expressed as mean ± SEM of at least four different experiments. Comparisons between groups were done by Student´s *t* test or Mann-Whitney´s test. Red letters and green asterisks represent statistical differences between different media in respiration and non-respiration, respectively. Fig (B): ^*a*^ *p* = 0.0009 (Student´s *t* test) relative to +Gln, *p<0.01 (Student´s *t* test)relative to +Gln. Fig (C): ^*a*^ *p* = 0.0008 (Mann-Whitney´s test) relative to +Serum, *p = 0.03 (Mann-Whitney´s test) relative to + Serum, Fig (D): ^*a*^ *p* = 0.0074 (Mann-Whitney´s test) relative to +Gln, *p = 0.0017 (Mann-Whitney´s test) relative to +Gln, Fig (E): ^*a*^ *p* = 0.001 (Mann-Whitney´s test) relative to +Serum, * *p* = 0.0008 (Mann-Whitney´s test) relative to +Serum.

In order to assess O_2_ utilization, 30 male or female worms were recovered from pre-incubation, as described in "*parasites*" section, and placed separately in 2 mL of the same "pre-incubation media" per chamber of a two-channel titration injection respirometer (Oxygraph-2k, Oroboros Instruments, Innsbruck, Austria). Data were recorded by using DatLab 5.0 software and measurements of O_2_ consumption were carried out at 37°C with sustained stirring at 750 rpm. In these experiments, O_2_ consumption by whole worms was recorded in basal conditions for 10 minutes (total OCR), followed by inhibition of ETS complex III activity by injecting 6 μg/mL antimycin A (AA) in each respirometer chamber. Then, respiration was left to proceed for 5–6 minutes. A typical O_2_ flux trace is depicted in Fig 1A. In order to assess the contribution of β-oxidation to worm respiration, 200 μM etomoxir (previously prepared in water) was added in basal conditions and then O_2_ fluxes recorded for about 10 minutes. Subsequently, 6 μg/mL AA was added to each respirometer chamber. In order to determine the mechanisms involved in O_2_ utilization in whole worms, we assumed the O_2_ consumption rate (OCR) sensitive to AA as respiration (R), the OCR sensitive to etomoxir as β-oxidation (β-ox) and finally, the OCR insensitive to AA as non-respiration (NR). O_2_ consumption rates resistant to etomoxir and sensitive to AA were considered as the contribution of oxidation of other substrates (OS) to respiration. To assess the contribution of oxidative phosphorylation (OXPHOS) and proton leak (LEAK) to basal respiration, we titrated oligomycin (5–45 μg/mL) in whole worms kept in HBSS containing 5.5 mM glucose and then O_2_ fluxes were recorded for about 10 minutes followed by addition of 6 μg/mL AA. We assumed the OCR sensitive to oligomycin as OXPHOS, while the LEAK OCR the one resistant to oligomycin and sensitive to AA.

### Mitochondrial enzyme activities

To determine mitochondrial enzyme activities, about 30 male or female worms were recovered from pre-incubation, as described in "*parasites*" section, and placed separately in 0.5 mL of hypotonic buffer (25 mM potassium phosphate and 5 mM MgCl_2_, pH 7.2) and then gently homogenized in a Potter-Elvehjem tissue grinder using a glass pestle. After homogenization, all samples were subjected to three liquid nitrogen-warm water bath freeze-thawing cycles. The preparation was maintained at 4°C throughout the procedure. CS activity was determined by measuring the reduction of 5,5-dithiobis (2-nitrobenzoic acid)(DTNB) according to the literature [90] with slight modifications. DTNB reduction was followed in a coupled reaction with coenzyme A and oxaloacetate. The reaction mixture (1 mL) containing, 0.25 mM DTNB, adult *S*. *mansoni* homogenate (12 μg protein), 0.3 mM acetyl CoA and 75 mM Tris-HCl, pH 8.0, was placed in a plastic cuvette and light absorption at 412 nm was assessed during 2 min (basal) at room temperature using a Shimadzu spectrophotometer UV/VIS 2450 (Shimadzu Scientific Instruments, Japan). Subsequently, CS reaction was started by adding 0.5 mM oxaloacetate to the cuvette and the rates of DTNB reduction were registered at 412 nm. CS activity was calculated by subtracting the rates of DTNB reduction induced by oxaloacetate from the basal using the molar extinction coefficient (ε = 13,600 M^−1^·cm^−1^) and expressed as nmol of reduced DTNB/min/mg protein. NADH:Cyt_c_ oxido-reductase activity of the electron transport system (ETS) was measured by following the increase in absorbance at 550 nm due to cytochrome *c* reduction [91]. The reaction containing 1 mL hypotonic buffer, 50 μM oxidized cytochrome *c*, 100 μg of protein from freeze-thawed worm homogenate samples and 1 mM KCN was initiated by adding 200 μM NADH and the increase in absorbance at 550 nm was monitored at room temperature in a Shimadzu spectrophotometer UV/VIS 2450 (Shimadzu Scientific Instruments, Japan) for about 5 minutes. Rotenone (5 μM) was added to inhibit complex I activity, which was considered as the rotenone-sensitive rate of cytochrome *c* reduction (ε = 18.5 mM^−1^ · cm^−1^). Cytochrome *c* oxidase activity was measured by following the decrease in absorbance due to the oxidation of ferrocytochrome *c*. The reaction containing 1 mL hypotonic buffer, 50 μM reduced cytochrome *c* was initiated by the addition of 100 μg of protein from freeze-thawed worm homogenate samples and the reduction in absorbance was measured at 550 nm. KCN (1 mM) was added to inhibit cytochrome *c* oxidase activity, which was considered as the cyanide-sensitive rate of cytochrome *c* oxidation.

### Hydrogen peroxide (H_2_O_2_) release

H_2_O_2_ production was assessed in whole intact worms by recovering individuals from pre-incubation in HBSS containing 5.5 mM Glucose or HBSS 5.5 mM Glucose + 5.5 mM Glutamine, as described in "*parasites*" section, and placing 30 male or female worms separately in 2 mL of the same pre-incubation media in a spectrofluorimeter cuvette. Parasite H_2_O_2_ production rates were determined by monitoring resorufin fluorescence due to the oxidation of 5 μM amplex red (Invitrogen, USA) in the presence of 200 μg/mL horseradish peroxidase (Sigma, USA) at room temperature using a Cary Eclipse spectrofluorimeter (Varian, USA) adapted with a continuous stirring device. Fluorescence was assessed using excitation and emission wavelengths of 530 nm and 590 nm, respectively. After each measurement, a standard curve of reagent grade H_2_O_2_ (Merck, Germany) was performed.

### Determination of total reduced thiol content

Total reduced thiol content was determined based on a previous method [92]. In this assay, 10 male or female worms were recovered from pre-incubation, as described in "*parasites*" section, and transferred separately to 24-well plates containing 500 μL RPMI 1640 without serum and phenol red (control), or 500 μL RPMI 1640 without serum and phenol red plus 10 μM menadione. The plates were incubated for 1h at 37°C and 5% CO_2_. After that, all the well content (media + worms) was collected and homogenized in a Potter-Elvehjem tissue grinder using a glass pestle at 4°C. Aliquots of 200 μL of homogenates were immediately mixed with 150 μL of 200 mM Tris buffer, pH 8.2, 10 μL of 10 mM DTNB (5, 5’-dithiobis-(2-nitrobenzoic acid) and 640 μL of 100% methanol. Sample blanks consisting of RPMI 1640 without serum, phenol red and DTNB were also prepared accordingly. All samples were incubated at room temperature (25°C) for 15 minutes, subsequently centrifuged at 3.000 x *g* for 15 minutes at 25°C, and supernatant light absorption was measured at 412 nm in a SpectraMax M5 plate reader (Molecular Devices, US). The content of total reduced thiols in homogenates was determined by subtracting the values found in the experimental group homogenates minus the values found in the sample blanks using the molar extinction coefficient (ε = 13,600 M^−1^·cm^−1^) to calculate the amount of reduced DTNB in each sample. Data for each sex was expressed as % total reduced thiols content in menadione treated worms relative to their respective controls.

### Quantification of intracellular oxidant levels by fluorescence microscopy

Worms recovered from pre-incubation in RPMI 1640 without serum and phenol red, as described in "*parasites*" section, were transferred separately to 96 wells plates and then incubated with 50 μM dihydroethidium (DHE) prepared in RPMI 1640 without serum and phenol red, at a density of 1 worm/well, in darkness for 20 minutes at 37°C and 5% CO_2_. Parasites were subsequently washed twice with 1 x PBS pH 7.4, re-suspended in 200 μL of 1 x PBS and whole body fluorescence images were captured in a stereo microscope (Olympus model MVX10) using excitation at 546 nm and emission at 590 nm. Images were analyzed by ImageJ/Fiji software [93], split into red, green and blue channels, but only the red channel images were used for quantification purposes. Masks were created on every image to define the worm body as the regions of interest, thus eliminating background signals. Average fluorescence intensity of masked images were registered and expressed as arbitrary fluorescence units.

### Viability assay

Worms recovered from pre-incubation in RPMI 1640 without serum and phenol red, as described in "*parasites*" section, were transferred separately to 48-well plates, at a density of 3 worms/well. Then, parasites were challenged with different concentrations of the pro-oxidant menadione (0–50 μM) in 500 μL RPMI 1640 without serum and phenol red for 2h at 37°C and 5% CO_2_. Antioxidant control experiments of worms exposed to 50 μM menadione and 1 mM *n–*acetyl cysteine (NAC) were also performed. Viability of worms was then assessed under a light microscope using the motility scale previously described [94]. The distinct parasite motile phenotypes were scored on a scale of 0–4 (4 = normally active; 3 = slow activity; 2 = minimal activity, occasional movement of head and tail; 1 = absence of motility apart from gut movements; 0 = total absence of mobility). Data were expressed as mean ± SEM of at least three different experiments

### Data and statistics

Data were presented as mean ± SEM values for all conditions. D´Agostino and Pearson normality test was performed to assess Gaussian distribution. Comparisons between groups were done by one-way ANOVA and *a posteriori* Tukey’s test for pair-wise comparisons. When appropriate, two-way ANOVA or unpaired Student’s t-tests or Mann-Whitney´s test were employed. Outlier values were excluded from data only when Gaussian distribution was achieved, using the online tool available at http://graphpad.com/quickcalcs/Grubbs1.cfm. Differences of *p*<0.05 were considered to be significant. Linear regression slopes comparisons between groups were conducted by using analysis of covariance (ANCOVA). All graphs and analyses were carried out by using the GraphPad Prism software version 5.00 for Windows (GraphPad Software, USA).

## Results and Discussion

### a) Nutritional requirements to sustain respiratory and non-respiratory O_2_ utilization in adult worms

Adult stages of *S*. *mansoni* live in the vertebrate bloodstream, where proteins, carbohydrates, lipids, and O_2_ are readily available and utilized by the parasites to sustain egg production [42–44] and energy demand through OXPHOS [45,46,50,51,56–59]. Since cells utilize O_2_ not only to couple nutrient oxidation to respiration, but also to allow reactions mediated by oxygenases [95–98], and reactive oxygen species (ROS) generation [15–17], in Fig 1 we assessed O_2_ utilization by whole intact adult unpaired females and males in media with four different nutrient compositions. Fig 1A shows a representative trace of O_2_ consumption rate (OCR) obtained from 30 intact *S*. *mansoni* males in RPMI 1640 media, where total OCR were registered at basal levels to define the total parasite O_2_ utilization. Then, 6 μg/mL antimycin a (AA) was injected to the respirometer chamber, and the resulting AA-sensitive component of total OCR was considered as "respiration", while the AA-insensitive, as "non-respiration" (Fig 1A). We observed that total OCR (S1A–S1D Fig), as well as the respiratory and non-respiratory components (Fig 1B–1E), were strikingly distinct depending on the nutrient availability. The lowest OCR values were obtained when 5.5 mM glucose was the only carbon source available for both parasite sexes (S1A, S1C,S1B and S1D Fig), while the highest ones were observed in the presence of RPMI+Serum (S1B, S1D, S1C and S1E Fig). Given that glutamine has previously reported to play a key energetic role in adult *S*. *mansoni* [56–59], our next step was to determine the contribution of this aminoacid to distinct O_2_ utilization pathways. Glutamine supplementation to HBSS + 5.5mM glucose, significantly increased total (S1A Fig), including the respiratory and non-respiratory OCR components in female worms (Fig 1B). Indeed, the presence of glutamine increased by 106% total OCR, 126% respiratory, and only 68% non-respiratory OCR when compared to glucose, indicating the preferential use of this aminoacid to sustain respiration in female worms. Supporting evidence from the literature demonstrate that glutamine metabolism does not affect glucose oxidation in adult worms [59]. In male parasites, glutamine significantly increased total (S1C Fig), as well as respiration and non-respiration OCR components relative to glucose (Fig 1D). Interestingly, the relative increases in OCR by glutamine supplementation were 73% in total, 62% in respiration, and 171% in non-respiration, indicating the remarkable contribution of glutamine metabolism to non-respiratory O_2_ utilization in male *S*. *mansoni*. Given that glutamine contributes to tricarboxylic acid cycle through α-ketoglutarate dehydrogenase complex (αKGDHC) activity, which represents an important site of mitochondrial hydrogen peroxide (H_2_O_2_) generation [99–101], it is possible that higher non-respiratory OCR induced by glutamine in male worms might result from increased ROS production through αKGDHC. Indeed, higher glutamate oxidation observed in male worms relative to females [57], provide supporting evidence for this proposal. In order to determine the contribution of other nutrients and vitamins to parasite O_2_ utilization, respirometry was also carried out in worms maintained in RPMI 1640 media in the absence or the presence of 10% fetal bovine serum (S1B and S1D Fig and Fig 1C and 1E). In general, absolute respiratory OCR values obtained in HBSS+Glc+Gln were quite similar as those from RPMI, suggesting that RPMI components other than glucose and glutamine have a negligible contribution to respiration in both parasite sexes. However, non-respiratory OCR in females were higher in RPMI relative to HBSS+Glc+Gln. Given that RPMI contains tyrosine, female tyrosinase activity might explain high non-respiratory OCR provided by this media, as this enzyme uses O_2_ to produce melanin allowing the formation and hardening of *Schistosoma* eggshell [88,102]. The highest parasite OCR values were achieved when serum was supplemented to RPMI 1640 media (S1 Fig), which might be due to increased supply of co-factors, nutrients and//or hormones present in serum that ultimately activate worm metabolism and O_2_ utilization [54,103,104]. Serum supplementation caused a relative increase by 190–195% in total OCR, 210–276% in respiration, and 56–173% in non-respiration in adult worms, indicating the remarkable contribution of serum to non-respiratory O_2_ utilization in male *S*. *mansoni*. However, since serum has a complex and undefined composition, we focused the next experiments in defined media without serum supplementation. As a result of respirometry analyses conducted in Fig 1 and S1 Fig, we observed that respiration represent the dominant mechanism of O_2_ utilization in *S*. *mansoni*, regardless the nutrient source available and parasite sex (S2 Fig). In female worms, the percent contribution of respiratory rates to total parasite O_2_ utilization were from 57% to 76%, while for non-respiration varied between 23% and 42%. In male parasites, the percent contribution of respiration to total OCR was higher than in females, ranging from 81% to 87%, while the opposite was observed for non-respiration, ranging from 12% to 18% (S1 Fig). Therefore, we conclude that glutamine and serum importantly contribute to respiratory and non-respiratory OCR in both parasites sexes, and that glutamine metabolism has a prominent contribution towards respiration in females and to non-respiration in males.

### b) Opposite sexual trends in O_2_ metabolism: high respiratory rates in male worms contrast with high non-respiratory rates in females independent of nutritional sources

**Fig 2:**
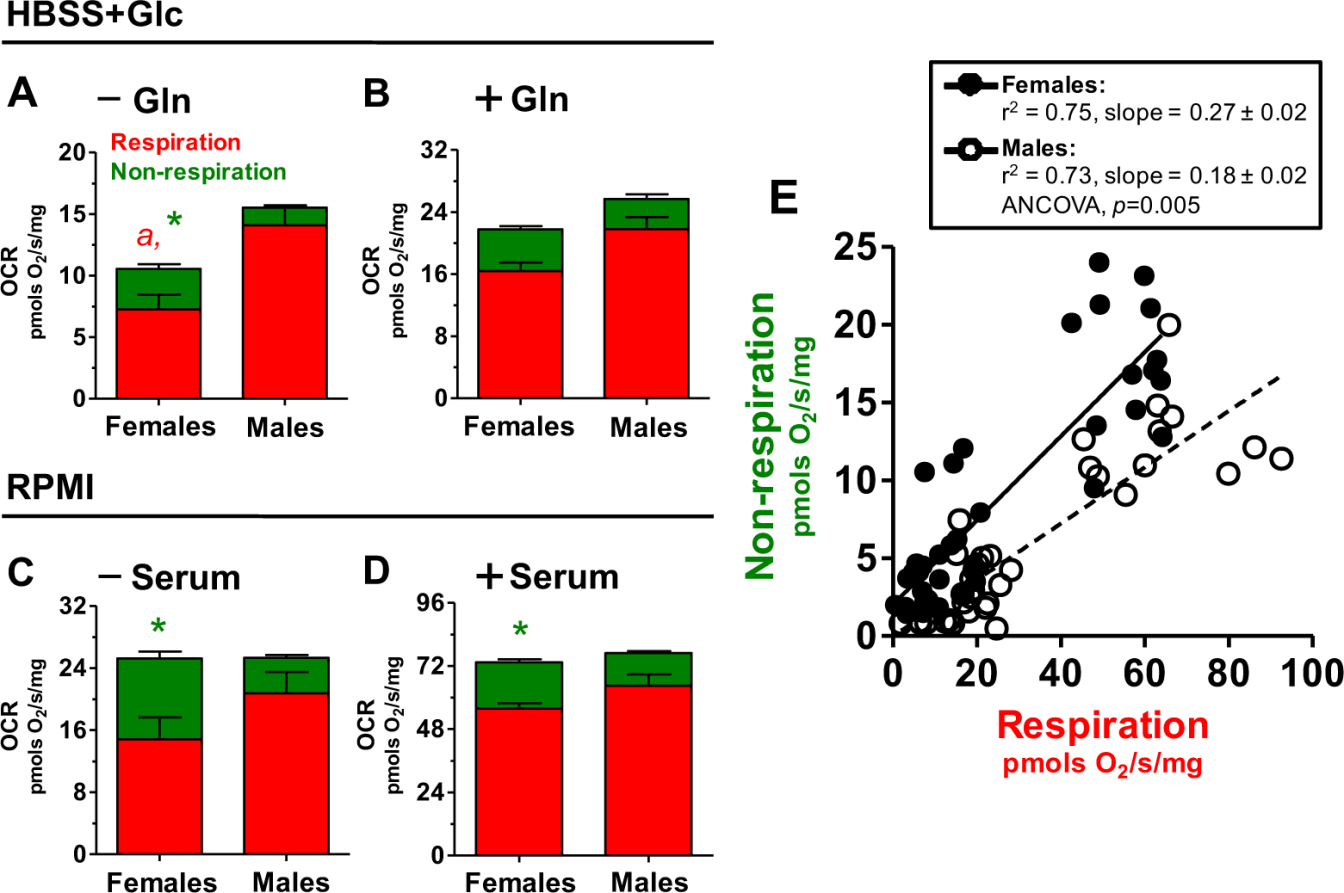
**Respiratory rates are higher in male worms, while non-respiratory O**_**2**_ **utilization rates are higher in females:** Comparative analyses of respiratory (red bars) and non-respiratory (green bars) OCR in adult female and male worms was determined by high resolution respirometry in media containing four different nutrient compositions as following: HBSS containing 5.5 mM glucose (HBSS+Glc) (A), HBSS containing 5.5 mM glucose plus 5.5 mM glutamine (+Gln) (B), RPMI 1640 (RPMI) (C), RPMI 1640 containing 10% fetal bovine serum (+Serum) (D). (E) Correlation analysis of non-respiratory and respiratory OCR in adult *S*. *mansoni* females (closed black circles) and males (open circles) determined in four media compositions, as described in Fig 1 and S1–S3 and S6 Figs. Data are expressed as mean ± SEM of at least four different experiments. Comparisons between groups were done by Student´s t-test, Mann-Whitney´s test or linear regression followed by ANCOVA analysis. Fig (A): ^*a*^ *p* = 0.0013 (Student´s *t* test) and * *p* = 0.0023 (Mann-Whitney´s test) relative to males. Fig (C): * *p* = 0.03 (Mann-Whitney´s test) relative to males. Fig (D): * *p* = 0.0025 (Mann-Whitney´s test) relative to males. Fig (E): ANCOVA *p* = 0.005.

Based on reported sexual differences in *Schistosoma* energy metabolism [46,51,56,76–83], and the distinct effects of nutrients on parasite O_2_ consumption shown in the present work (Fig 1 and S1 Fig), we then compared O_2_ utilization pathways between unpaired female and male parasites in different media compositions (Fig 2 and S2 and S3 Figs). We observed that males exhibited slightly higher OCR values in glucose (Fig 2B) and RPMI media (Fig 2C) compared to females. However, the general trend observed was that respiration in male worms was slightly higher than females (Fig 2A, 2B, 2C and 2D), reaching significance only when parasites were maintained in 5.5 mM glucose as the only nutritional source (*p*<0.005, Fig 2A). These observations are consistent with higher expression and activity of glycolytic pathway enzymes in male worms relative to females [78,105–107]. Also, these data agree with similar total O_2_ consumption rates observed among sexes [47], despite CO_2_ production from glucose, glutamate and other aminoacids were higher in male worms compared to females in two *Schistosoma* species [52,57]. In this sense, the activity of tricarboxylic acid (TCA) cycle enzymes aconitase, malate dehydrogenase and isocitrate dehydrogenase were reportedly higher in male *Schistosoma* compared to females [54,105–107]. This strongly indicates that male *S*. *mansoni* worms have improved capacity to mediate nutrient oxidation and respiration, which is substantiated by the data shown in Fig 2A–2D. Indeed, mitochondria isolated from males exhibit higher respiratory rates using glutamate, malate and succinate as substrates compared to females (S1 Methods and S4 Fig), despite the relative increases in OCR provided by ADP were quite similar in both sexes (140% and 127% for females and males, respectively). Also, the contribution of oxidative phosphorylation (OXPHOS) to basal respiration do not differ among sexes, while the proton leak OCR have a trend to be higher in males (S5 Fig). Interestingly, assessment of non-respiratory O_2_ utilization rates reveals an opposite sexual trend, where females exhibited significantly higher absolute OCR values than males, in most experimental conditions (Fig 2A, 2C and 2D). Indeed, the relative contribution of non-respiratory pathways to total O_2_ utilization in females was significantly higher (23–42% of total OCR) than in males (12–18% of total OCR), regardless the nutritional sources available (S3A, S3B, S3C and S3D Fig). Fig 2E shows a correlation analysis of absolute rates of O_2_ utilization through respiratory and non-respiratory pathways for female and male worms in all nutritional conditions tested in Fig 2. We observed that the slope of the curve generated for female worms was significantly steeper than that for males (*p* = 0.006), indicating that O_2_ utilization through non-respiratory mechanisms is higher in females, while respiratory rates were higher in male worms. Conceivably, increased activity of distinct oxygenases reportedly to be functional in *Schistosoma* [88,102,108–110], or cellular ROS production by the own parasite metabolism might explain high non-respiration OCR values observed in females. In conclusion, an opposite sexual trend in O_2_ utilization was observed in adult *Schistosoma* parasites, in a way that high respiratory rates in male worms contrast with high non-respiratory rates in females independent of nutritional sources.

### c) Similar contribution of β-oxidation to respiration among parasite sexes

Although morphology and motility of adult worms hardly change in anoxic conditions, egg production and viability are drastically affected [43]. Interestingly, fatty acids provided *in vitro* were able to support egg laying and development [111], and recent evidence demonstrated that lipolysis and β-oxidation are absolutely required to allow oogenesis [42].Given the distinct sexual trends in nutrient utilization observed in the present work (Figs 1 and 2, S1–S3 Figs), and the importance of fatty acid oxidation to *S*. *mansoni* physiology [42], we then investigated the contribution of β-oxidation to respiration in both parasite sexes kept in RPMI+serum, by means of pharmacological intervention of carnitine palmitoyltransferase I (CPT1) provided by etomoxir [112].We observed that blockage of fatty acid transport to mitochondria by etomoxir had a consistent reduction in respiration in both parasite sexes. Quantification of the etomoxir-sensitive OCR demonstrated that the extent by which β-oxidation contributes to respiratory rates is roughly similar among parasite sexes (31% and 29% in female and male parasites, respectively) (S6 Fig). Interestingly, these data agree with quite similar CD36 expression levels among parasite sexes [78], which mediates cholesteryl ester uptake and might participate in β-oxidation in adult worms [113]. Although the data shown in S6 Fig are consistent with the key role of lipolysis and β-oxidation to O_2_ utilization and egg production in female worms [42], the biological significance of mitochondrial fatty acid oxidation in male worms remain elusive. Two possibilities should be considered in this context: *i) motor activity*: given the abundance of muscle fibers in adult male worms, the energy demand to sustain muscle contraction might be partially achieved through β-oxidation, similar to vertebrate muscle fibers [114,115]; *ii) reproduction*: although the contribution of fatty acid oxidation to spermatozoa motility is quite limited in other models [116], this process may play an energetic role in male *S*. *mansoni* spermatozoa. Finally, no gender differences were observed in the capacity to oxidize fatty acids, suggesting that mitochondrial content and/or the enzyme machinery directly involved in this process are also comparable among parasite sexes.

### d) Increased complex I-III, but not complex IV activity in female worms, favors electron delivery to mitochondria

**Fig 3:**
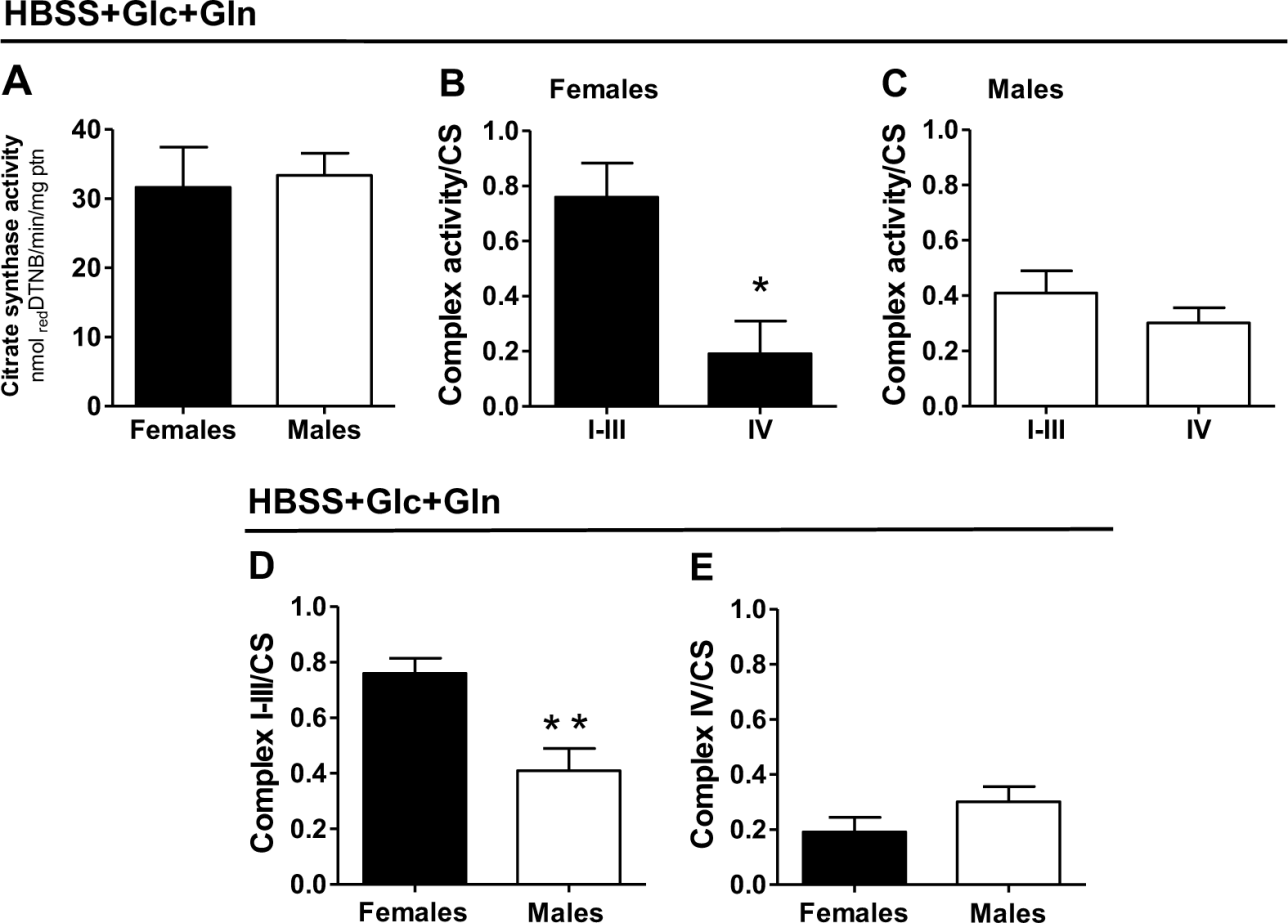
Higher electron transport chain complex I-III activity in adult *S*. *mansoni* females despite similar mitochondrial content among sexes. Mitochondrial enzymes activities were measured in homogenates of adult females (black bars) and males (white bars) previously maintained in HBSS containing 5.5mM glucose plus 5.5 mM glutamine (HBSS+Glc+Gln). (A) Citrate synthase (CS) activity was assessed following the increase in light absorption at 412 nm, while complex I-III and complex IV (B and C) activities were determined through reduction (for complex I-III) or oxidation (for complex IV) of cytochrome *c* at 550 nm. Comparative analyses of complex I-III (D) or complex IV (E) activities in females (black bars) and males (white bars) homogenates, normalized by their respective CS activities. Data are expressed as mean ± SEM of at least five different experiments. Comparisons between groups were done by Mann-Whitney´s test. Fig (B): * *p*<0.01. Fig (D): * *p*<0.05.

Given that male parasites exhibit higher activity of some TCA cycle enzymes [54,105–107], and higher respiratory rates compared to females (Fig 2 and S3 Fig), we then investigated whether mitochondrial content and electron transport system (ETS) complex activities might explain these sexual differences. S7 Fig and Fig 3A show that citrate synthase (CS) activity was quite similar among tested media and parasite sexes, indicating that mitochondrial content per protein mass was similar in female and male worms independent of nutritional sources available. Indeed, CS activities measured in our experimental conditions were quite close in absolute values to those reported earlier in the literature [105]. Interestingly, we observed that complex I-III activity was higher than complex IV specifically in females (Fig 3B and 3C), regardless the media composition (Fig 3D and S9 Fig). This result contrasts with the recent transcriptome analyses of adult *Schistosoma* [78] which show that seven distinct complex I subunits were significantly more expressed in male worms compared to females. Since in our experiments, NADH-induced cytochorme *c* reduction depends on ubiquinone levels and complex III, it might be that these components compensate the reduced expression of complex I subunits to allow high complex I-III activity in female worms. Regarding complex IV activity, no differences were observed among parasite sexes and media composition (Fig 3E and S9D–S9F Fig). Of note, all enzyme activities shown in Fig 3B–3E were expressed normalized by CS, indicating that increased complex I-III activity observed in female worms is not a consequence of higher mitochondrial content. Therefore, higher complex I-III relative to complex IV activity in female worms would provide a more efficient mechanism for electron entry to ETS compared to males. Overall, the data shown in Fig 3 and S7–S9 Figs indicate that improved capacity to mediate nutrient oxidation and respiration observed in male worms (Fig 2A and 2E and S3 Fig) is not a consequence of higher mitochondrial content or to improved ETS complex activities, but rather to other mechanisms, such as efficient substrate transport and/or metabolism through specific TCA cycle enzymes [54,105–107]. Conceivably, limited mitochondrial pyruvate carrier activity in females may explain lower respiratory rates compared to males (Fig 2 and S3 Fig), even though exhibiting higher complex I-III activity (Fig 3D and S9A–S9C Fig). In addition, despite the similar capacity to oxidize fatty acids agrees with comparable CS and complex IV activities among sexes, the differences observed in female and male O_2_ utilization (Fig 2 and S3 Fig) cannot be explained by these mechanisms. On the other hand, higher complex I-III activity observed in female worms, contrast with their lower respiratory rates relative to male worms, suggesting that increased electron flux in female mitochondria might have a distinct fate than fully reduce O_2_ to water through complex IV. Therefore, it is possible that higher electron delivery to ETS through complex I-III activity in females would favor mitochondrial electron leak and ROS production, which is consistent with reduced respiratory rates and higher O_2_ utilization through non-respiratory pathways (Fig 2 and S3 Fig).

### e) Female worms exhibit higher levels of intracellular oxidants relative to males

**Fig 4:**
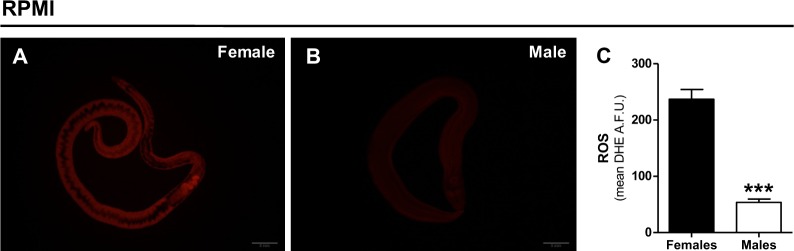
Intracellular oxidant levels are higher in females. The levels of intracellular oxidants were assessed in whole female (A) and male (B) worms incubated in RPMI 1640 without phenol red and 50 μM DHE. Fluorescence stereomicroscope images were collected for whole individual worms, the mean fluorescence intensity was quantified and expressed as arbitrary units (C). Data are expressed as mean ± SEM of at least seventeen different experiments. Comparisons between groups were done by using Student´s t- test. Fig (C): ***p<0.001.

Despite the knowledge on parasite antioxidant mechanisms [11–14,65–72], and their relevance as a valuable chemotherapeutic target [25,65,68–70,74,75], direct evidence of endogenous ROS production by adult *Schistosoma* worms, and its biological significance, remains so far elusive. Of note is the sexual dimorphism that adult parasites exhibit in many distinct aspects, including the energy [47,52,57,79–84] and redox metabolism [67,78,85–87], with potential implications for rational and sex-specific drug design for schistosomiasis. In this sense, redox homeostasis was assessed in adult worms by evaluating the intracellular levels of oxidants through the use the fluorescent probe dihydroethidium (DHE) [117]. Both female and male *S*. *mansoni* were kept in RPMI 1640 media without phenol red and images were acquired in a fluorescence stereoscope. Fig 4 shows representative DHE fluorescence images of both female (Fig 4A) and male (Fig 4B) worms, which stained quite homogeneously throughout the worm body in both sexes. In female worms, reduced fluorescence in the gut lumen could be due to interference of erythrocytes remnants and hemozoin crystals on light excitation or emission. Indeed, fluorescence intensity was clearly higher in female worms (Fig 4A), indicating higher levels of oxidants relative to male *S*. *mansoni* (Fig 4B). The differences observed in DHE staining are not due to limited probe uptake in males, as incubation of intact worms with an unrelated dye (DAPI) revealed the exact opposite trend (higher fluorescence in males compared to females) (S1 Methods and S10 Fig). These data are in agreement with the higher non-respiratory O_2_ utilization observed in female worms relative to males, irrespective of the nutritional sources available (S3 Fig). Fig 4C shows the quantification of DHE fluorescence intensities in both parasite sexes, and confirms that oxidant levels in females were strikingly higher than in male worms. However, it is important to consider that the assessment of DHE fluorescence through imaging, as a proxy to detect intracellular ROS levels, has several limitations [117], and should be interpreted with caution and supported by alternative methods. Thus, in order to directly assess total ROS production, we next quantified H_2_O_2_ production rates by whole parasites using the amplex red method.

### f) Sexual preferences in substrate utilization defines endogenous reactive oxygen species production in adult *S*. *mansoni*

**Fig 5:**
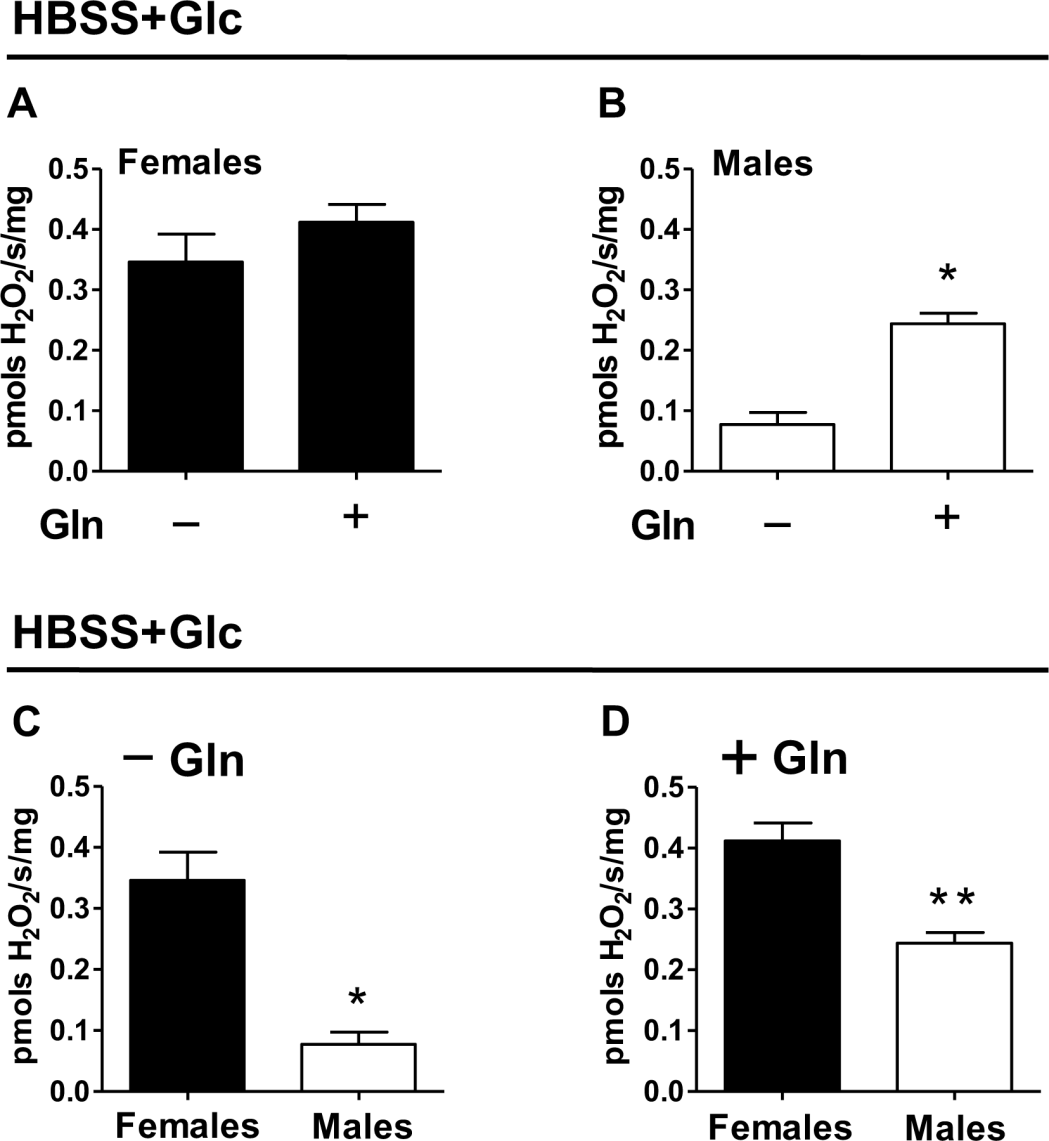
Higher H_2_O_2_ production rates in females is independent of nutritional source. H_2_O_2_ production rates were quantified in adult intact females (black bars) and males (white bars) previously maintained in HBSS containing 5.5 mM glucose (HBSS+Glc), or HBSS containing 5.5 mM glucose plus 5.5 mM glutamine (HBSS+Glc+Gln), by following the fluorescence of Amplex red oxidation. (A) Females and (B) males H_2_O_2_ production rates promoted by different media composition. (C) Comparative analyses of H_2_O_2_ production rates among parasite sexes in HBSS+Glc (C) or HBSS+Glc+Gln (D). Data are expressed as mean ± SEM of at least four different experiments. Comparisons between groups were done by Mann-Whitney test. Fig (B): * *p*<0.05. Fig (C): * *p*<0.05. Fig (D): * *p*<0.01.

Cellular ROS production is mediated by distinct mechanisms [15–17], with quite specific substrate dependencies [15]. Beyond their central energetic role, glucose and glutamine metabolism also regulate redox homeostasis, through the pentose phosphate pathway [118], and glutaminase-mediated lactate production [119] respectively. In both cases, these substrates contribute to maintain redox balance by generating reducing potential as NADPH to allow buffering of antioxidant defenses. Although glutamine metabolism directly participates on glutathione synthesis, reducing ROS production and limiting redox imbalance [120], in specific conditions this aminoacid contributes to cellular ROS production [121–123]. Thus, in order to assess how nutrient mediate endogenous ROS production in adult parasites, we next determined the rates of H_2_O_2_ production in whole intact *S*. *mansoni* worms kept under two distinct nutritional conditions: 5.5 mM glucose and 5.5 mM glucose + 5.5 mM glutamine. Fig 5A shows that H_2_O_2_ production rates by female worms were high and similar in both media composition (19% higher in glucose + glutamine relative to glucose), suggesting that glucose metabolism plays a dominant role in female ROS production. The rates of H_2_O_2_ production were clearly lower in male worms (Fig 5B) compared to females, especially when glucose was the only substrate available. Interestingly, glutamine supplementation caused a significant increase (*p*<0.02) in H_2_O_2_ generation rates relative to glucose in male worms (215% higher in glucose + glutamine relative to glucose), suggesting that this aminoacid plays a major role in male *S*. *mansoni* ROS production (Fig 5B). These data agree with a 117% increase in non-respiratory O_2_ utilization relative to glucose provided by glutamine in male worms (Fig 2B). Therefore, adult parasites exhibit clear sexual substrate preferences for H_2_O_2_ generation (glucose in females and glutamine in males). Direct comparisons among sexes revealed that H_2_O_2_ production rates were significantly higher in female worms, regardless the available substrates. Indeed, the rates of H_2_O_2_ formation in females were 4.4 and 1.7 times higher than in males in glucose and glucose+glutamine, respectively (Fig 5C and 5D). This result fully agrees with the higher oxidant levels found in female worms revealed by DHE staining (Fig 4). Also, the sexual differences in H_2_O_2_ production are in line with the higher non-respiratory O_2_ utilization observed in female worms relative to males, irrespective of the nutritional sources available (Fig 2E).

**Fig 6:**
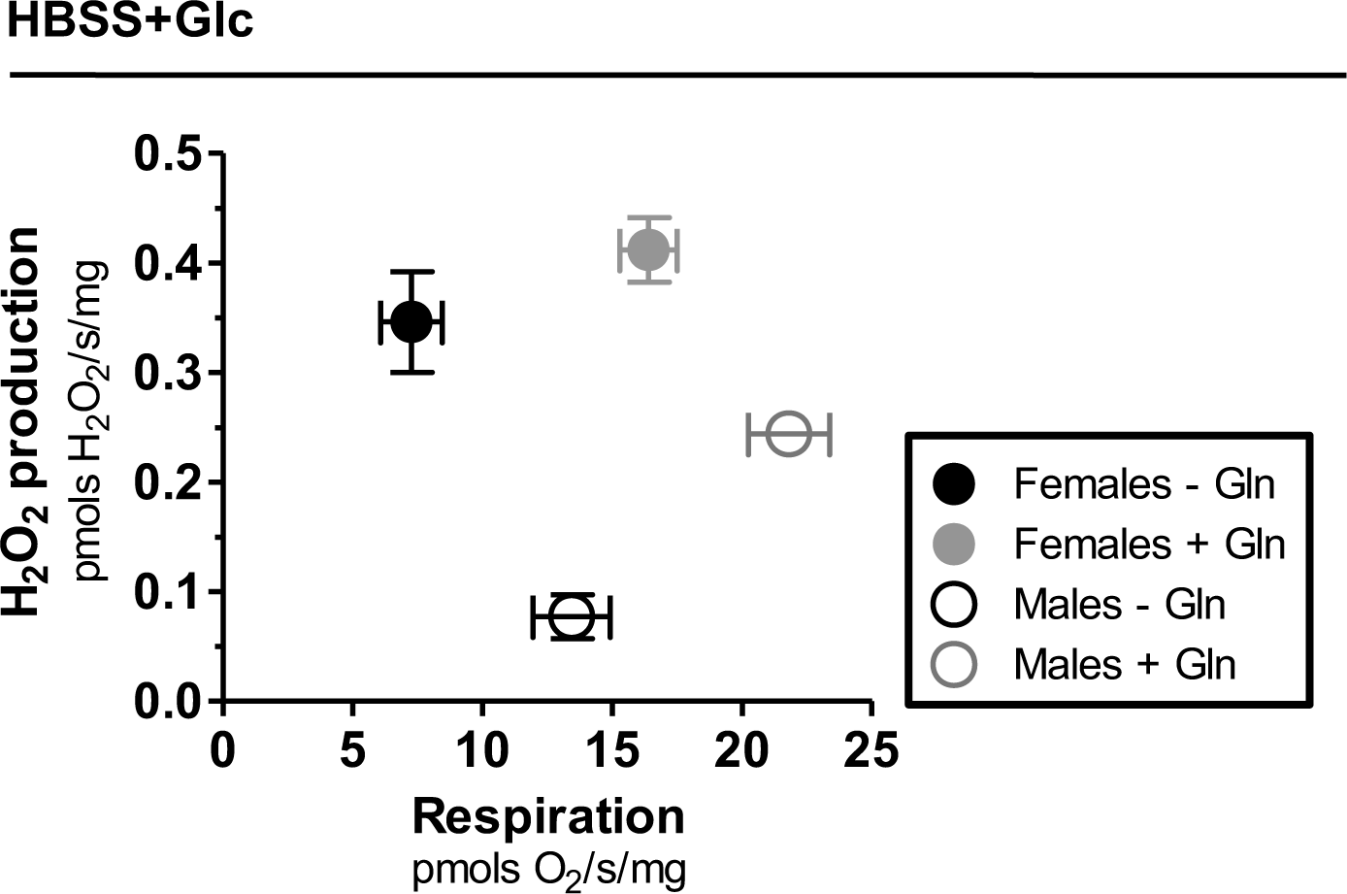
Low respiratory rates in females are linked to increased H_2_O_2_ production. Respiratory OCR values and H_2_O_2_ production rates were determined in females (closed circles) and males (open circles) in HBSS+5.5 mM Glc (black circles) and HBSS+5.5 mM Glc+5.5 mM Gln (gray circles). Data were derived from Figs 1 and 5 are expressed as mean ± SEM of at least four different experiments.

Fig 6 shows comparative analyses of H_2_O_2_ generation rates in female and male worms kept under glucose and glucose + glutamine (Fig 5A and 5B), by their respective respiratory rates (Fig 1B and 1D). We observed that lower respiratory rates in females were associated to higher H_2_O_2_ production in both substrate conditions, compared to males. Interestingly, the increase in respiratory rates provided by glutamine in females hardly affected H_2_O_2_ production, suggesting that glutamine utilization is not a limiting factor for ROS production. This is consistent with our observation that glutamine supplementation in female worms increased OCR mostly through respiration rather than non-respiratory pathways (Fig 1B). Conceivably, despite glutamine oxidation promoted higher respiratory rates through the glutaminase-TCA cycle-OXPHOS path in females, the resulted increase in mitochondrial electron flow would not be followed by higher ROS production at ETS, as the antioxidant capacity would increase H_2_O_2_ detoxification by increasing glutathione and NAPDH production, which would avoid redox imbalance [119–120]. On the other hand, glutamine caused a proportional increase in both parameters in male worms, suggesting that increased glutamine oxidation and mitochondrial electron flow result in higher ROS production, as the capacity to synthesize glutathione and NAPDH from glutamine would be limited in male worms. Alternatively, αKGDH complex structure might be different in male worms, facilitating electron leak and ROS production at this site [99–101], without compromising its activity and respiratory rates. In conclusion, these observations suggest that H_2_O_2_ production in adult *S*. *mansoni* worms is sustained by sexual preferences in substrate utilization towards glucose in females, and glutamine in male worms.

### g) Female worms are more resistant to a pro-oxidant challenge

**Fig 7:**
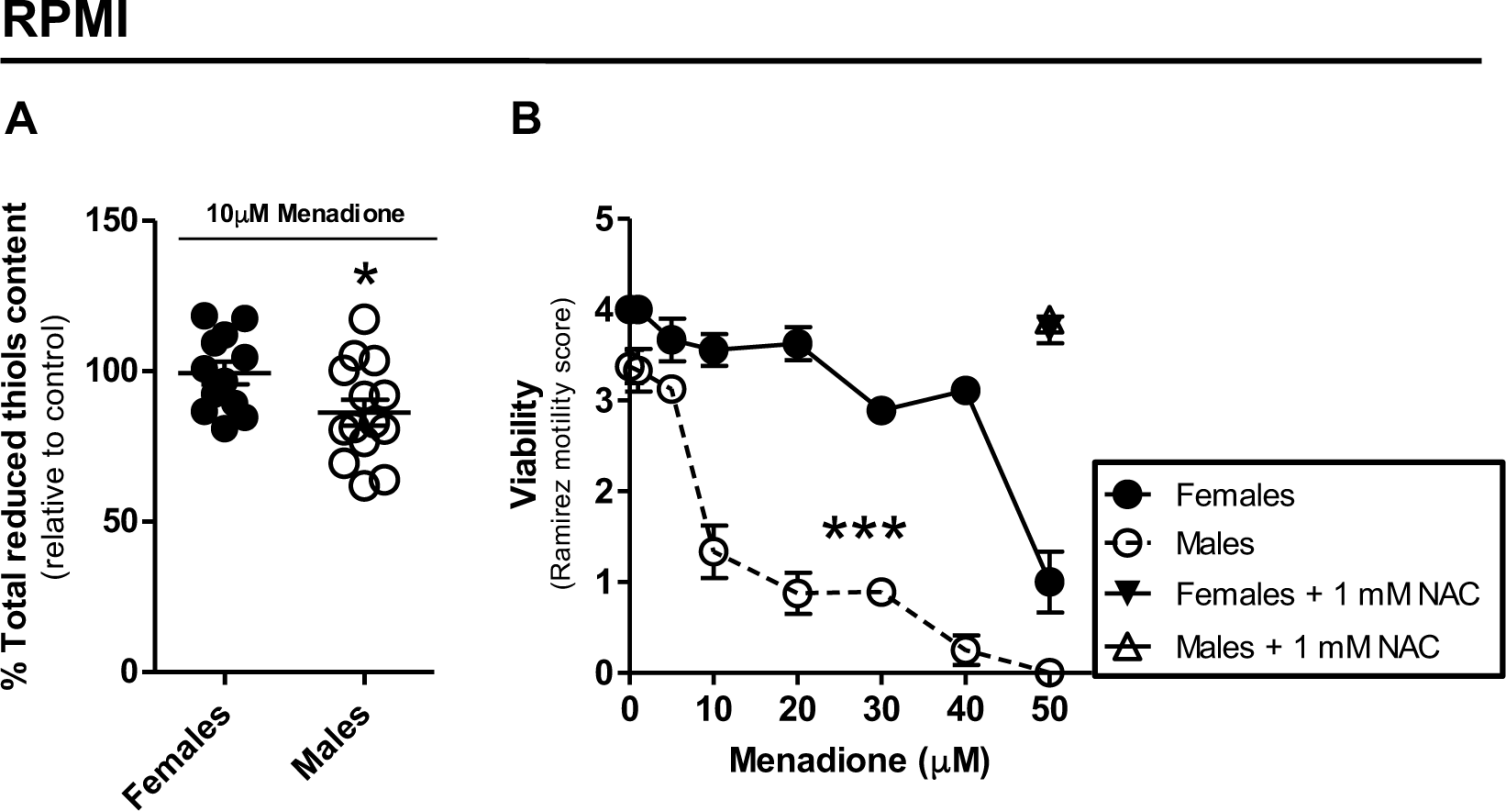
Female worms are more resistant to a pro-oxidant challenge. (A) Total reduced thiol contents were determined by light absorption at 412 nm in females (closed circles) or males (open circles) homogenates after 1h pre-incubation of intact worms with 10 μM menadione in RPMI 1640. (B) Viability of females (closed circles) or males (open circles) was determined by light microscopy after 2h pre-incubation of intact worms with different concentrations of menadione in RPMI 1640 using a previously reported motility scale [91]. Females (closed triangle) or males (open triangle) viability incubated for 2h with 50 μM menadione and 1 mM n-acetylcysteine (NAC) were also determined. Data were expressed as mean ± SEM of four (A) or three (B) different experiments. Comparisons between groups were done by Student´s t-test (A) and two-way ANOVA and *a posteriori* Bonferroni (B). Fig (A): * *p*<0.05. Fig (B): *** *p*<0.001.

A number of observations support the concept that sustained mild oxidative stress conditions trigger protective antioxidant responses in different models [124–134]. These adaptive responses not only confer protection to redox insults [125,126], but also provide other beneficial outputs, such as increased motor capacity [133], insulin sensitivity [134], improved glucose homeostasis [134], and longevity [124,127,128,130,133]. Similar protective antioxidant responses were also reported for *S*. *mansoni* [135–139]. For example, the expression of parasite redox defense mechanisms was induced by either redox imbalance conditions or by chemotherapeutic agents [135–137], while hyperbaric O_2_ therapy in *S*. *mansoni*-infected hamsters increased the number of recovered worms and parasite eggs trapped within tissues [138]. Curiously, the antioxidant n-acetylcysteine (NAC) potentiated the effect of arthemether on parasite killing in a murine schistosomiasis model [139]. These evidence indicate that adult *S*. *mansoni* worms are able to regulate ROS production and/or their antioxidant defenses in order to cope with oxidant insult, thus avoiding parasite death. Although total oxidant levels and H_2_O_2_ generation were consistently higher in female worms (Figs 4–6), it remains unknown whether endogenous ROS production is associated to protective adaptive responses in adult *S*. *mansoni*. In order to address this issue, we acutely exposed adult worms to menadione, a known pro-oxidant that stimulates endogenous ROS production and intracellular thiol oxidation [140–144]. Interestingly, Fig 7A shows that the content of total reduced thiols in female worms were not affected after 1h challenge with 10 μM menadione, while significant reductions (~13%)were observed male worms. When parasites were acutely exposed with different concentrations of menadione, we observed a dose-dependent reduction in viability of both parasite sexes, assessed by their motility [91] (Fig 7B). Strikingly, we observed that female worms were significantly more resistant to menadione challenge than male worms (Fig 7B). Indeed, motility of female worms hardly changed up to 20 μM menadione treatment, whereas strong reductions in this parameter were observed in male worms within this concentration range. Also noteworthy, the loss of parasite viability was mediated by redox imbalance, as this effect was completely blunted in both parasite sexes by co-treatment with 50 μM menadione plus 1 mM NAC. Together, these results indicate that female worms exhibit higher resistance to oxidative stress conditions generated by endogenous ROS production, which agrees with their higher content of antioxidant defenses compared to male parasites [67,78,85–87].

## Conclusions and Final Remarks

**Fig 8:**
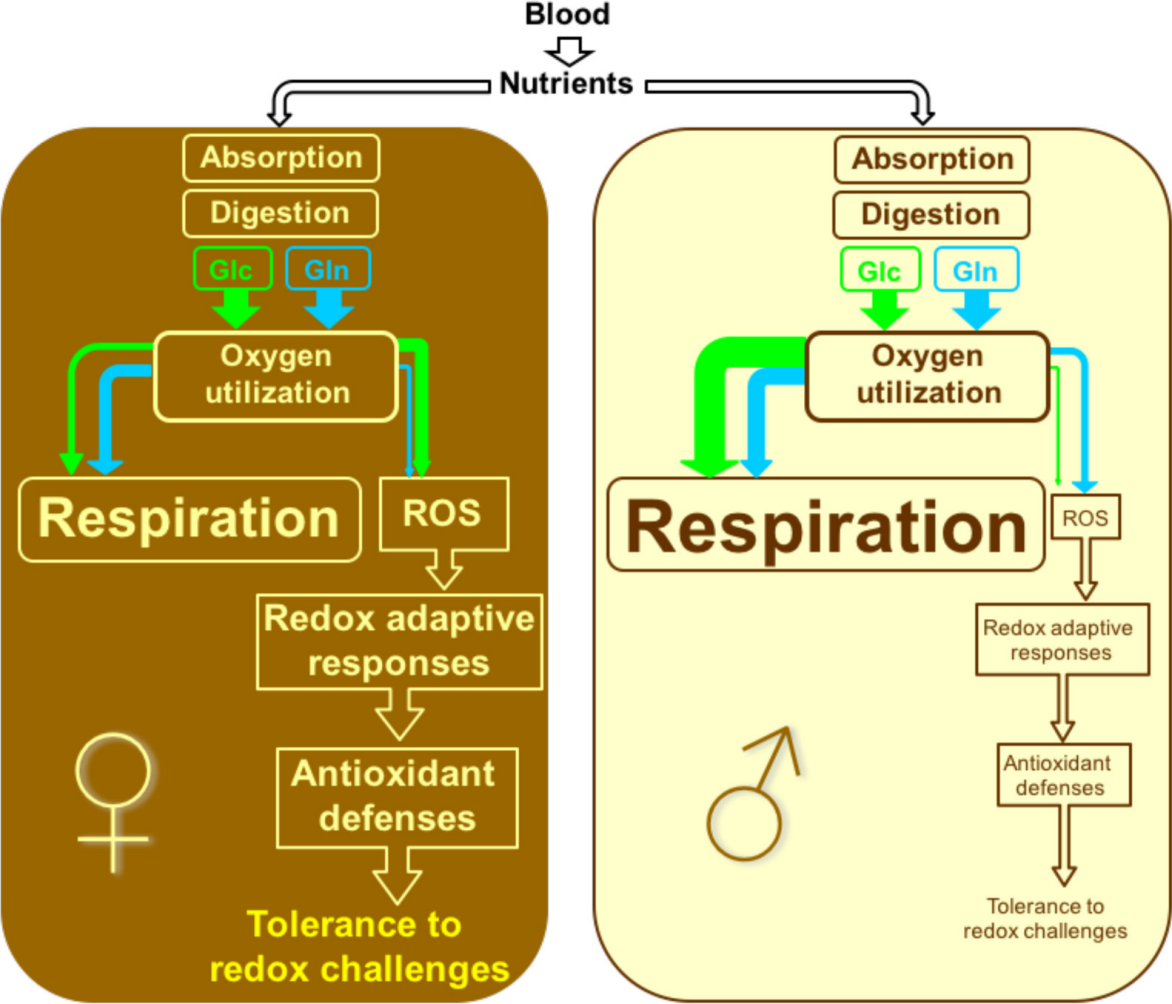
Schematic representation on how sexual preferences in nutrient utilization regulate O_2_ consumption and ROS generation in adult *S*. *mansoni* and the potential implications to parasite redox biology. Brown and light yellow boxes represent female and male worms, respectively. Nutrients derived from the bloodstream are absorbed by the parasites through either transtegumental transport or through the gut upon digestion by luminal enzymes. The present work focused on the contribution of glucose (green) and glutamine (blue) on O_2_ utilization pathways and the consequences to energy and redox metabolism. Nutrient oxidation is carried out by different enzymes of the parasite energy metabolism, releasing electrons, which are ultimately utilized by O_2_ demanding processes as a final acceptor to allow: *i)* respiration at cytochrome *c* oxidase activity (Respiration), which is directly connected to energy metabolism; *ii)* reactive oxygen species (ROS) generation that take place at multiple cellular sites. ROS produced by the parasite triggers redox adaptive mechanisms, essentially involving antioxidant defenses, which finally mediate tolerance to endogenous and/or physiological redox challenges. Sexual differences in pathways described in the present work are highlighted by the sizes of arrows, fonts and boxes. Female *S*. *mansoni* exhibit higher contribution of glutamine to respiration, compared to glucose, whereas in male worms, glucose metabolism has a major role to support respiration, compared to glutamine. As a whole, male *S*. *mansoni* exhibit higher O_2_ consumption rates by means of respiration than females, while O_2_ utilization through non-respiratory pathways was higher in females, which was mostly linked to glucose metabolism. In males, non-respiratory O_2_ utilization was essentially provided by glutamine metabolism. Non-respiratory O_2_ consumption rates are directly connected to H_2_O_2_ and ROS production, exhibiting the same substrates dependencies among sexes. H_2_O_2_ production was mostly sustained by glucose oxidation in females, while in males, glutamine was the preferred substrate to generate ROS. Conceivably, increased ROS formation in females activate redox adaptive responses, mediating the build-up of antioxidant defenses, and allowing improved tolerance to oxidative stress.

In the present work, we demonstrate that in adult *S*. *mansoni* worms sexual preferences in nutrient utilization regulate endogenous parasite O_2_ consumption and ROS generation, which might differently contribute to redox biology among parasite sexes. A summary of the results presented here is schematically depicted in Fig 8. Blood-derived nutrients are absorbed through worm tegument or gut, and metabolized to their fundamental units. Glucose and glutamine are differentially oxidized through respiratory and non-respiratory pathways in adult worms, demanding distinct rates of O_2_ utilization among nutrients and parasite sexes. Glutamine metabolism plays a dominant role to sustain respiratory pathways in female worms, while glucose is the preferential substrate for this purpose in males. Interestingly, the opposite trend is observed in both non-respiratory O_2_ utilization and ROS formation among sexes, since glucose is the preferential substrate to generate ROS in females, while in male worms, glutamine prevails. Overall, respiratory rates are higher in males compared to females, while the rates of ROS production are higher in females. To cope with higher ROS levels generated endogenously by their own metabolism, redox adaptive mechanisms are more intensely activated in females than in males, mediating the build-up of antioxidant defenses, which ultimately confer higher tolerance to oxidative stress conditions. Therefore, we suggest that the higher levels of antioxidant defenses [67,78,85–87], and tolerance to redox challenges observed in female worms, seem to be a consequence of their higher endogenous ROS production generated by the parasite metabolism. Given that schistosomiasis clinically manifests by the inflammatory reaction triggered by antigens released by parasite eggs deposited in host tissues, targeting redox adaptive response mechanisms in adult worms might reveal an interesting venue for the development of new classes of schistosomicidal agents.

A number of evidence support the notion that ROS are key intracellular messengers, mediating adaptive stress response mechanisms, and promoting a number of positive physiological and cytoprotective effects [124–134,145–149]. For instance, improved stress resistance and longevity of *Caenorhabditis elegans* worms are induced by a variety of stress signals that have in common their ability to stimulate mitochondrial ROS formation [124,127,128,130,133,146,148,149]. Also, strains of the causative agent of Chagas´ disease (*Trypanosoma cruzi*), with increased antioxidant defenses are more resistant to redox insults and are more virulent, suggesting a relationship between redox balance and infectivity [150]. In this regard, our group demonstrated that *T*. *cruzi* bloodstream trypomastigote forms were strikingly resistant to oxidative challenges, but were able to generate higher levels of H_2_O_2_ than epimastigote forms [151]. Mechanistically, increased complex II-III and reduced complex IV activity observed in bloodstream forms, create an electron bottleneck facilitating electron leakage to produce O_2_^•¯^and H_2_O_2_, which seems to provide an oxidative pre-conditioning conferring protection against redox imbalance conditions. Indeed, despite the acute exposure to high levels of ROS is unquestionably cytotoxic, chronic mild oxidative stress conditions clearly promote beneficial outputs for many different models [127–133,146]. Conceivably, adult *S*. *mansoni* worms are able to respond differently to oxidative insults through hormetic responses, in a way that female redox homeostasis would be mostly controlled by the detoxification rates rather than their generation, whereas in males the opposite would be true. In this regard, *S*. *mansoni* worms are capable of inducing an adaptive response against redox challenges, by increasing the expression of antioxidant [136,152,153], and repair enzymes [152].

We are aware of the potential limitations inherent of the experimental approach employed in the present work. Firstly, all processes investigated here were carried out with worm s*ex vivo*, in O_2_ tensions much higher than those found in mesenteric veins of the vertebrate host. Potentially, the exposure to high O_2_ tensions might overwhelm the worm´s antioxidant capacity, exacerbating their O_2_ utilization for both energy transduction and ROS generation. Secondly, given that pairing regulates physiological aspects of adult *S*. *mansoni* [82–84], it might be possible that the conjugal biology might also be determinant to parasite redox homeostasis. An example in this regard is the higher expression of antioxidant enzymes in paired *S*. *japonicum* females compared to unpaired ones [154,155]. However, it is important to emphasize that the experimental approach employed here to interrogate the sexual trends in energy and redox metabolism of adult worms would be nearly impossible to achieve using paired worms, as the particular metabolic features for one sex would be masked by the other. Despite the growing need to understand basic aspects of schistosomiasis, the specific sites and pathways (i.e. mitochondria, NADPH oxidase, peroxisomes) involved on endogenous ROS production remain elusive. The data presented here indicate that unpaired adult *S*. *mansoni* worms exhibit sexual preferences in nutrient utilization that regulate O_2_ consumption and ROS generation pathways which might provide differential tolerance to oxidative stress among parasite sexes. Future research will provide insights over key aspects described here, especially those related to the mechanisms that mediate redox adaptive responses in *S*. *mansoni* females, and the potential implications for parasite survival.

## Supporting Information

S1 Fig Nutrients regulate total O_2_ utilization in adult worms. Comparative analyses of total O_2_ consumption of adult female (A, black bars) and male (B, white bars) worms determined in four different nutrient compositions as following: HBSS + 5.5 mM glucose, HBSS + 5.5 mM glucose + 5.5 mM glutamine, RPMI 1640, or RPMI 1640 + serum. Data are expressed as mean ± SEM of at least four different experiments. Comparisons between groups were done by Mann-Whitney´s (letters) or Student´s *t* tests (symbols). Fig (A): ^*a*^ *p* = 0.0025 relative to +Gln, Fig (B): ^*a*^ *p*<0.004 relative to +Serum, Fig (C): ^*a*^ *p* = 0.014 relative to +Gln, Fig (D): ^*a*^ *p*<0.004 relative to +Serum, Fig (E): * *p*<0.015 relative to males.
(TIF)

S2 Fig Relative contributions of respiratory and non-respiratory O_2_ utilization in adult worms in different nutritional conditions. The relative contribution of respiration (red bars) and non-respiration (green bars) to total OCR of adult female (A) and male (B) worms were determined in four different nutrient compositions as following: HBSS + 5.5 mM glucose, HBSS + 5.5 mM glucose + 5.5 mM glutamine, RPMI 1640, or RPMI 1640 + serum. Data shown are mean ± SEM of at least four different experiments.
(TIF)

S3 Fig Sexual differences in relative contribution of respiratory and non-respiratory O_2_ utilization among diets in adult worms. Comparative analyses of respiratory (red bars) and non-respiratory (green bars) OCR in adult female and male worms was determined by high resolution respirometry in media containing HBSS + 5.5 mM glucose (A), HBSS + 5.5 mM glucose + 5.5 mM glutamine (B), RPMI 1640 (C), or RPMI 1640 + serum (D). Data are expressed as mean ± SEM of at least four different experiments. Comparisons between groups were done by Student´s t-test or Mann-Whitney´s test. Red letters and green symbols over bars represent statistical differences between sexes in respiration and non-respiration, respectively. Statistical symbols are relative to males. In all comparisons, Mann-Whitney´s test was applied. Fig (A): ^a^ *p*<0.0001, * *p*<0.0001. Fig (B): ^b^ *p*<0.05, ** *p*<0.05. Fig (C): ^b^ *p*<0.05, ** *p*<0.05. Fig (D): ^c^ *p*<0.002, ^#^ *p*<0.002.
(TIF)

S4 Fig Isolated mitochondria from male worms exhibit higher respiratory capacity. Typical traces of O_2_ consumption in isolated mitochondria from female (A) and male (B) worms, using 10 mM glutamate + 1 mM malate (Glu-Mal) and 10mM succinate (Suc) as substrates (See S1 Methods). To assess state 2 respiration, the OCR induced by Glu+Mal+Suc was subtracted from that resistant to AA, while the state 3 was determined after addition of 1 mM ADP. (C) Comparison of respiratory states 2 and 3 demonstrate higher OCR in male mitochondria. Data are expressed as mean ± SEM of n = 1 for females and n = 2 for males.
(TIF)

S5 Fig Respiration linked to oxidative phosphorylation is quite similar between sexes. O_2_ consumption rates (OCR) of intact adult *S*. *mansoni* worms were determined by high resolution respirometry in media containing four different nutrient compositions as following: HBSS containing 5.5 mM glucose. (A) Representative OCR trace obtained from 30 intact *S*. *mansoni* males. The OCR coupled to oxidative phosphorylation was assessed by inhibiting ATP synthase with multiple injections of oligomycin. The OCR sensitive to 25 μg/mL oligomycin was used to determine OXPHOS (B), while the residual OCR insensitive to oligomycin, but inhibited by AA, was considered as “proton leak” (C). Data are expressed as mean ± SEM of four different experiments.
(TIF)

S6 Fig Limited contribution of mitochondrial β-oxidation to respiration in adult *S*. *mansoni* worms is not distinct among sexes. The contribution of lipid metabolism through beta-oxidation (β-ox, blue bars) and by other substrates (OS, pink bars) were determined in adult female and male worms by high resolution respirometry in media containing RPMI 1640 + serum. To determine the contribution of mitochondrial β-oxidation to respiration, basal OCR was measured followed by addition of 200 μM etomoxir to each respirometer chamber. The etomoxir-sensitive component of the respiration was considered as β-ox, while the remaining etomoxir-insensitive one (OS), as derived from oxidation of substrates other than lipids.
(TIF)

S7 Fig Similar mitochondrial content among parasite sexes regardless the nutrient sources. Citrate synthase activity was measured in adult female (black bars) and male (white bars) worms kept in three different nutrient compositions, as following: HBSS + 5.5 mM glucose, HBSS + 5.5 mM glucose + 5.5 mM glutamine, or RPMI 1640 + serum. Data shown are mean ± SEM of at least five different experiments.
(TIF)

S8 Fig Complex I-III is specifically higher in female worms regardless the nutrient sources. Complex I-III (C I-III) and complex IV (C IV) activities were measured in adult female (black bars) and male (white bars) worms kept in three different nutrient compositions, as following: HBSS + 5.5 mM glucose, HBSS + 5.5 mM glucose + 5.5 mM glutamine, or RPMI 1640 + serum. Data shown are mean ± SEM of at least five different experiments. Comparisons between groups were done by Student´s t-test (A) or Mann-Whitney´s test (B and C). Fig (A): * *p*<0.01 relative to C I-III. Fig (B): * *p*<0.02 relative to C I-III. Fig (C): * *p*<0.01 relative to C I-III.
(TIF)

S9 Fig Female complex I-III activity is higher than in males regardless the nutrient sources. Complex I-III (C I-III) and complex IV (C IV) activities were measured in adult female (black bars) and male (white bars) worms kept in three different nutrient compositions, as following: HBSS + 5.5 mM glucose, HBSS + 5.5 mM glucose + 5.5 mM glutamine, or RPMI 1640 + serum. Data shown are mean ± SEM of at least five different experiments. Comparisons between groups were done by Student´s t-test (A) or Mann-Whitney´s test (B and C). Fig (A): * *p*<0.001 relative to females. Fig (C): * *p*<0.02 relative to females.
(TIF)

S10 Fig DAPI uptake is higher in males. Worms were cultured for 24 h in RPMI + serum, and subsequently incubated in RPMI 1640 without phenol red supplemented with 1 μg/mL DAPI for 20 minutes at 37°C and 5% CO_2_ (See S1 Methods). Fluorescence microscopy images were collected for whole individual worms, the average fluorescence intensity was quantified and expressed as arbitrary units. Data are expressed as mean ± SEM of at least three different experiments. Comparisons between groups were done by using Mann-Whitney´s test. ***p<0.001.
(TIF)

S1 Methods (DOCX)

## Abbreviations

NTD: neglected tropical diseases
O_2_: molecular oxygen
ROS: reactive oxygen species
H_2_O_2_: hydrogen peroxide
O_2_^•^−^^: superoxide
CS: citrate synthase
ΔΨ_m_: mitochondrial membrane potential
OXPHOS: oxidative phosphorylation
TCA: tricarboxylic acid
Prx: peroxiredoxin
GSH: reduced glutathione
Trx: thioredoxin
TGR: thioredoxin glutathione reductase
Glc: glucose
Gln: glutamine
OCR: oxygen consumption rate
R: respiration
NR: non-respiration
OS: other substrates
DTNB: 5,5-dithiobis(2-nitrobenzoic acid)
DHE: dihydroethidium
NAC: *n–*acetyl cysteine
SEM: standard error of mean
AA: antimycin a
αKGDHC: α-ketoglutarate dehydrogenase complex
CPT1: carnitine palmitoyltransferase I
ETS: electron transport system
