# Supplementary figures and images for: Sexual Preferences in Nutrient Utilization Regulate Oxygen Consumption and Reactive Oxygen Species Generation in Schistosoma mansoni: Potential Implications for Parasite Redox Biology
Source: PLoS One. 2016 Jul 5;11(7):e0158429. doi: 10.1371/journal.pone.0158429 (PMC4933344; doi:10.1371/journal.pone.0158429)

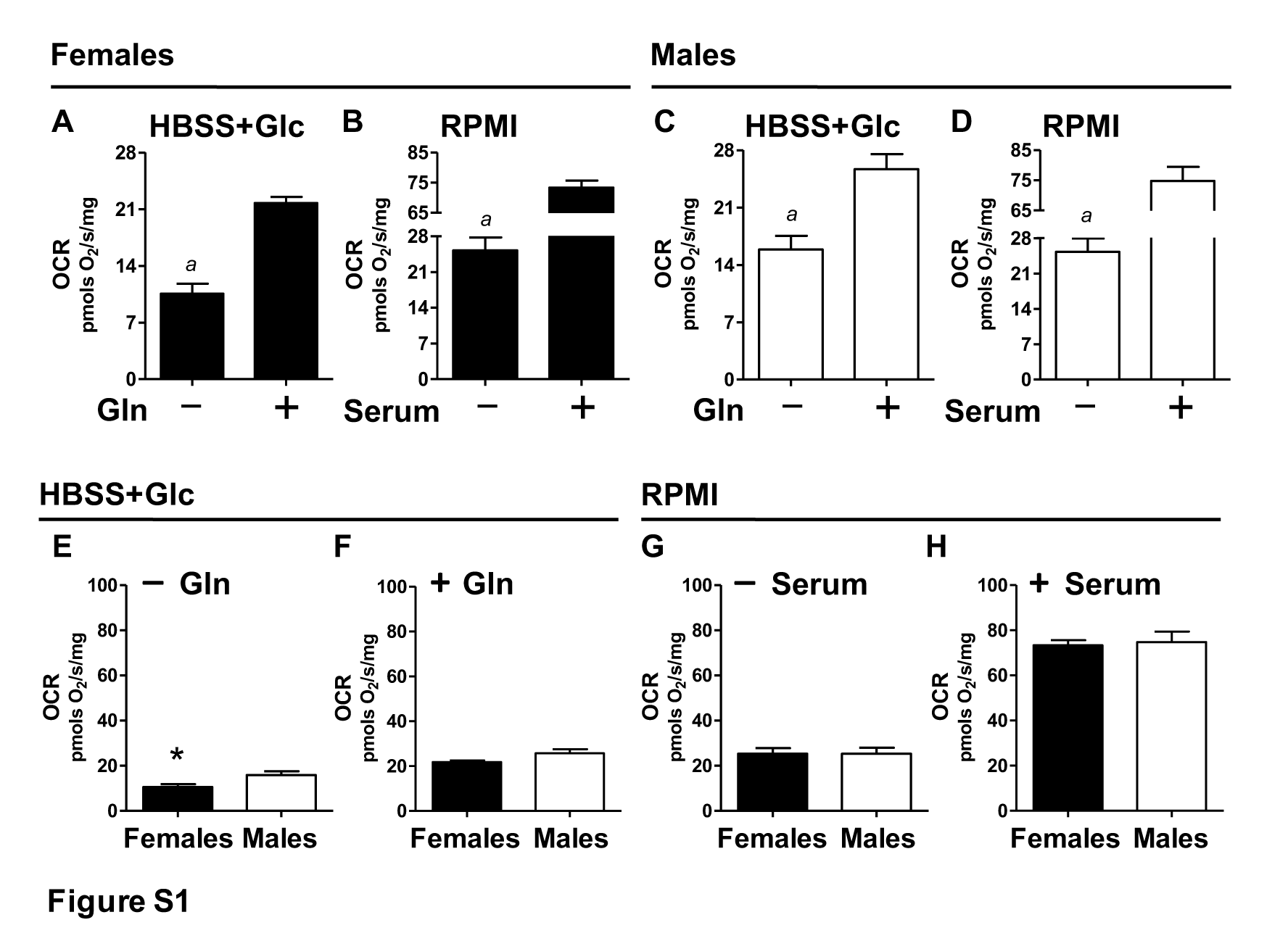

Supplement: S1 Fig — Comparative analyses of total O2 consumption of adult female (A, black bars) and male (B, white bars) worms determined in four different nutrient compositions as following: HBSS + 5.5 mM glucose, HBSS + 5.5 mM glucose + 5.5 mM glutamine, RPMI 1640, or RPMI 1640 + serum. Data are expressed as mean ± SEM of at least four different experiments. Comparisons between groups were done by Mann-Whitney´s (letters) or Student´s t tests (symbols). Fig (A): a p = 0.0025 relative to +Gln, Fig (B): a p<0.004 relative to +Serum, Fig (C): a p = 0.014 relative to +Gln, Fig (D): a p<0.004 relative to +Serum, Fig (E): * p<0.015 relative to males. (TIF) [file pone.0158429.s001.tif]

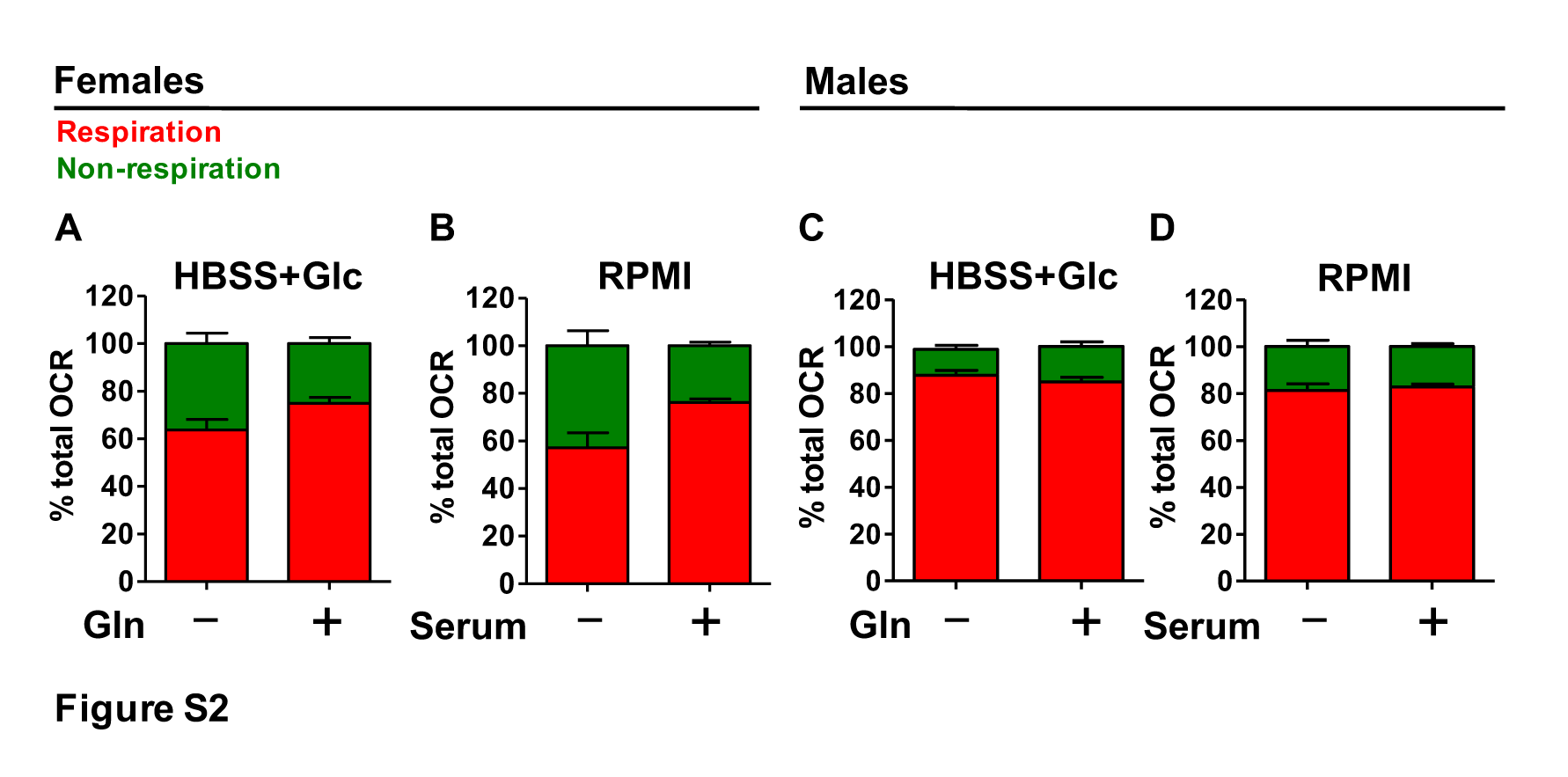

Supplement: S2 Fig — The relative contribution of respiration (red bars) and non-respiration (green bars) to total OCR of adult female (A) and male (B) worms were determined in four different nutrient compositions as following: HBSS + 5.5 mM glucose, HBSS + 5.5 mM glucose + 5.5 mM glutamine, RPMI 1640, or RPMI 1640 + serum. Data shown are mean ± SEM of at least four different experiments. (TIF) [file pone.0158429.s002.tif]

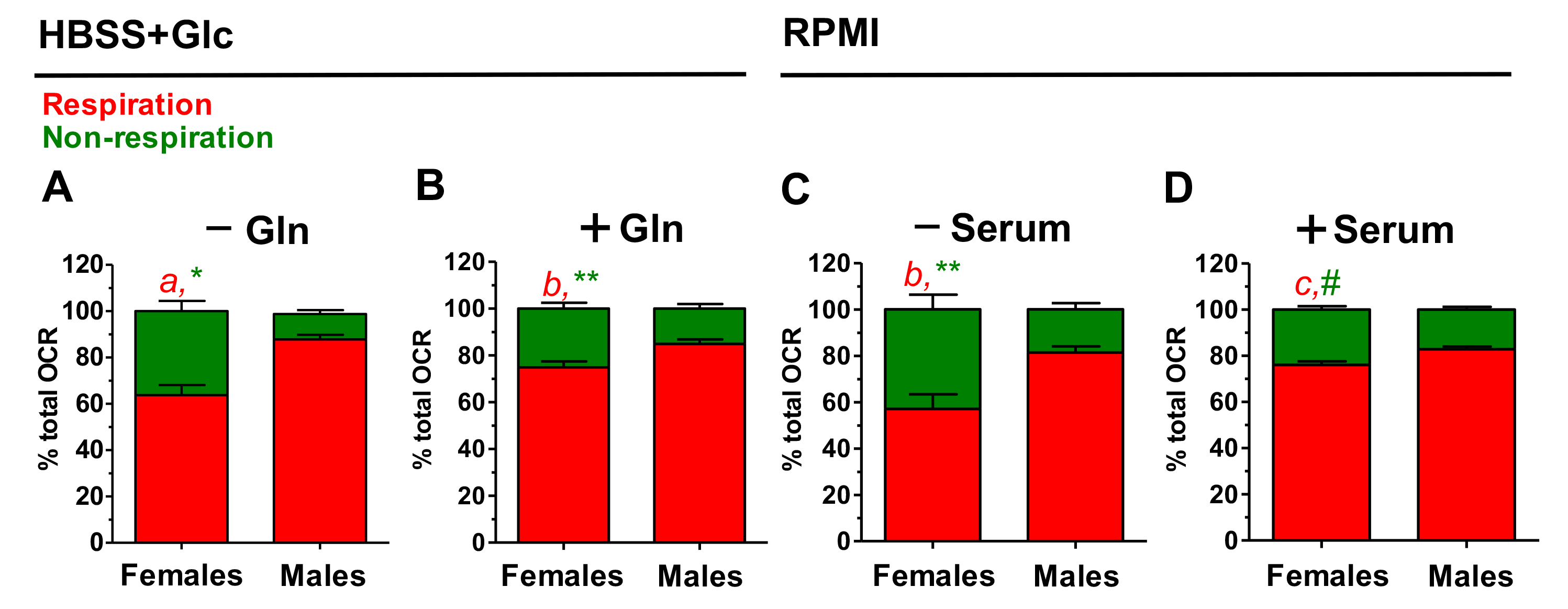

Supplement: S3 Fig — Comparative analyses of respiratory (red bars) and non-respiratory (green bars) OCR in adult female and male worms was determined by high resolution respirometry in media containing HBSS + 5.5 mM glucose (A), HBSS + 5.5 mM glucose + 5.5 mM glutamine (B), RPMI 1640 (C), or RPMI 1640 + serum (D). Data are expressed as mean ± SEM of at least four different experiments. Comparisons between groups were done by Student´s t-test or Mann-Whitney´s test. Red letters and green symbols over bars represent statistical differences between sexes in respiration and non-respiration, respectively. Statistical symbols are relative to males. In all comparisons, Mann-Whitney´s test was applied. Fig (A): a p<0.0001, * p<0.0001. Fig (B): b p<0.05, ** p<0.05. Fig (C): b p<0.05, ** p<0.05. Fig (D): c p<0.002, # p<0.002. (TIF) [file pone.0158429.s003.tif]

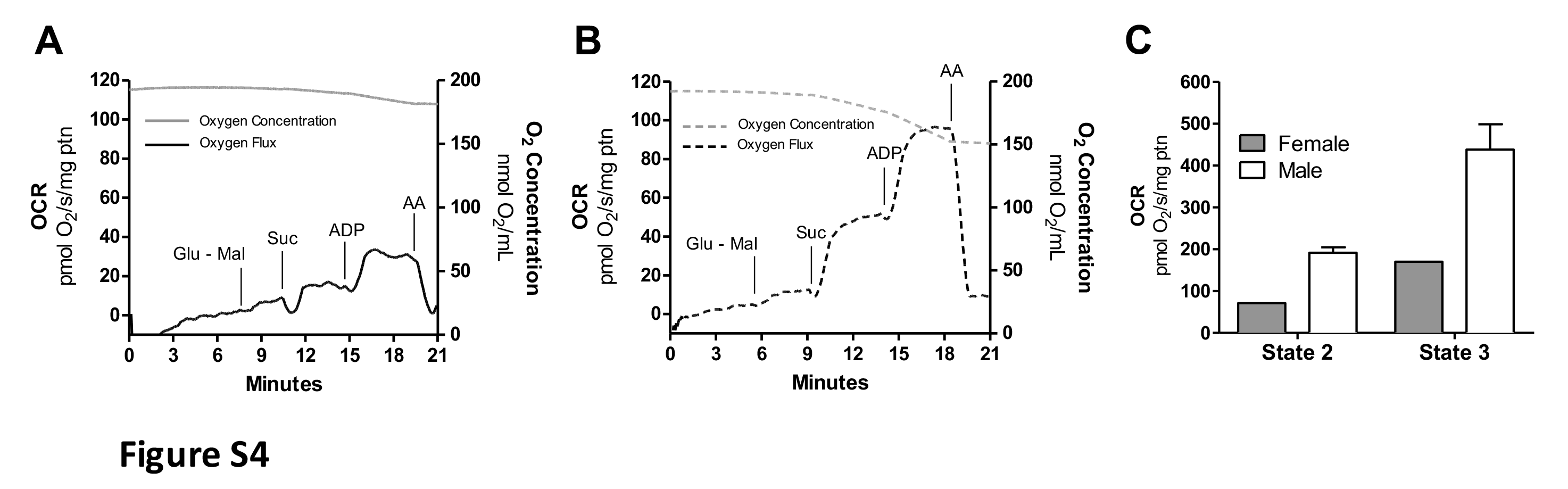

Supplement: S4 Fig — Typical traces of O2 consumption in isolated mitochondria from female (A) and male (B) worms, using 10 mM glutamate + 1 mM malate (Glu-Mal) and 10mM succinate (Suc) as substrates (See S1 Methods). To assess state 2 respiration, the OCR induced by Glu+Mal+Suc was subtracted from that resistant to AA, while the state 3 was determined after addition of 1 mM ADP. (C) Comparison of respiratory states 2 and 3 demonstrate higher OCR in male mitochondria. Data are expressed as mean ± SEM of n = 1 for females and n = 2 for males. (TIF) [file pone.0158429.s004.tif]

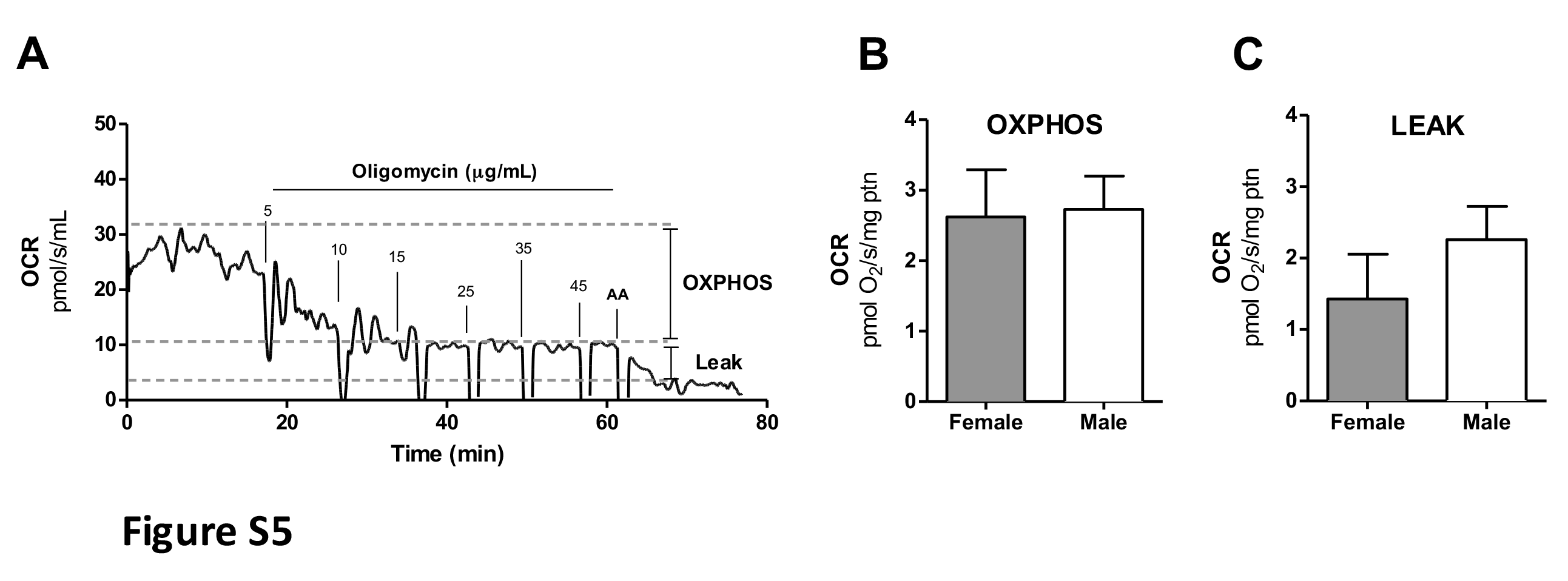

Supplement: S5 Fig — O2 consumption rates (OCR) of intact adult S. mansoni worms were determined by high resolution respirometry in media containing four different nutrient compositions as following: HBSS containing 5.5 mM glucose. (A) Representative OCR trace obtained from 30 intact S. mansoni males. The OCR coupled to oxidative phosphorylation was assessed by inhibiting ATP synthase with multiple injections of oligomycin. The OCR sensitive to 25 μg/mL oligomycin was used to determine OXPHOS (B), while the residual OCR insensitive to oligomycin, but inhibited by AA, was considered as “proton leak” (C). Data are expressed as mean ± SEM of four different experiments. (TIF) [file pone.0158429.s005.tif]

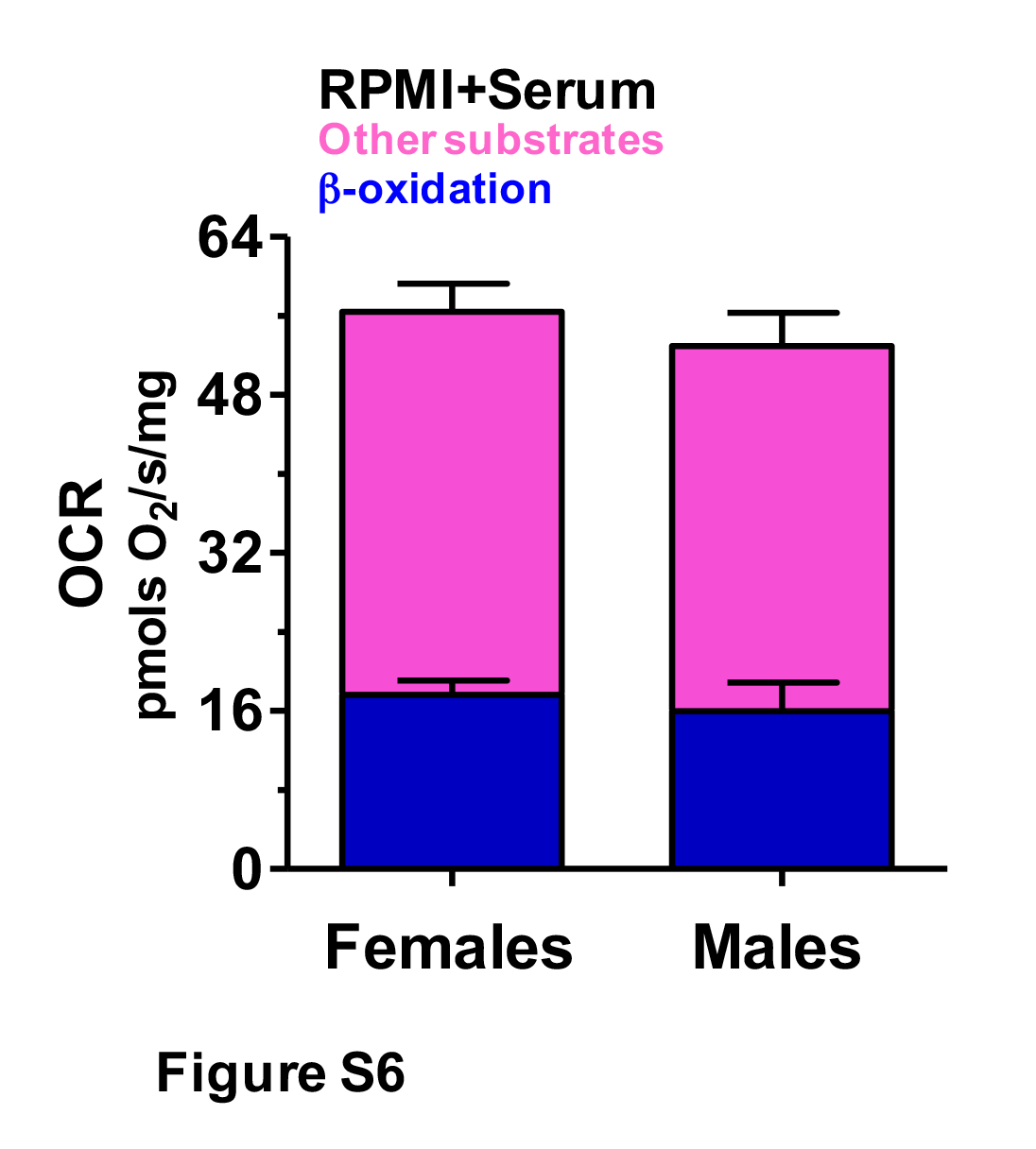

Supplement: S6 Fig — The contribution of lipid metabolism through beta-oxidation (β-ox, blue bars) and by other substrates (OS, pink bars) were determined in adult female and male worms by high resolution respirometry in media containing RPMI 1640 + serum. To determine the contribution of mitochondrial β-oxidation to respiration, basal OCR was measured followed by addition of 200 μM etomoxir to each respirometer chamber. The etomoxir-sensitive component of the respiration was considered as β-ox, while the remaining etomoxir-insensitive one (OS), as derived from oxidation of substrates other than lipids. (TIF) [file pone.0158429.s006.tif]

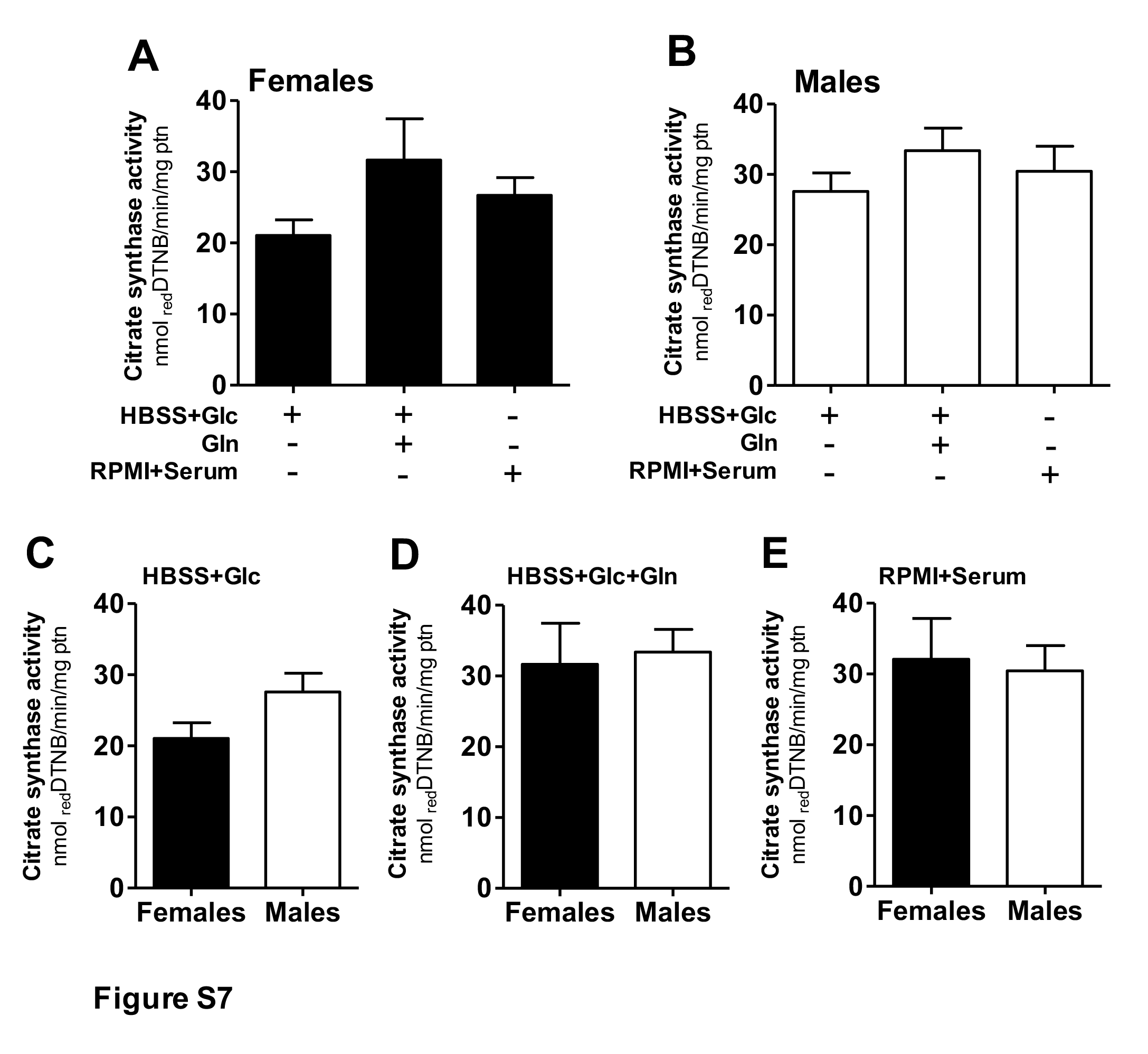

Supplement: S7 Fig — Citrate synthase activity was measured in adult female (black bars) and male (white bars) worms kept in three different nutrient compositions, as following: HBSS + 5.5 mM glucose, HBSS + 5.5 mM glucose + 5.5 mM glutamine, or RPMI 1640 + serum. Data shown are mean ± SEM of at least five different experiments. (TIF) [file pone.0158429.s007.tif]

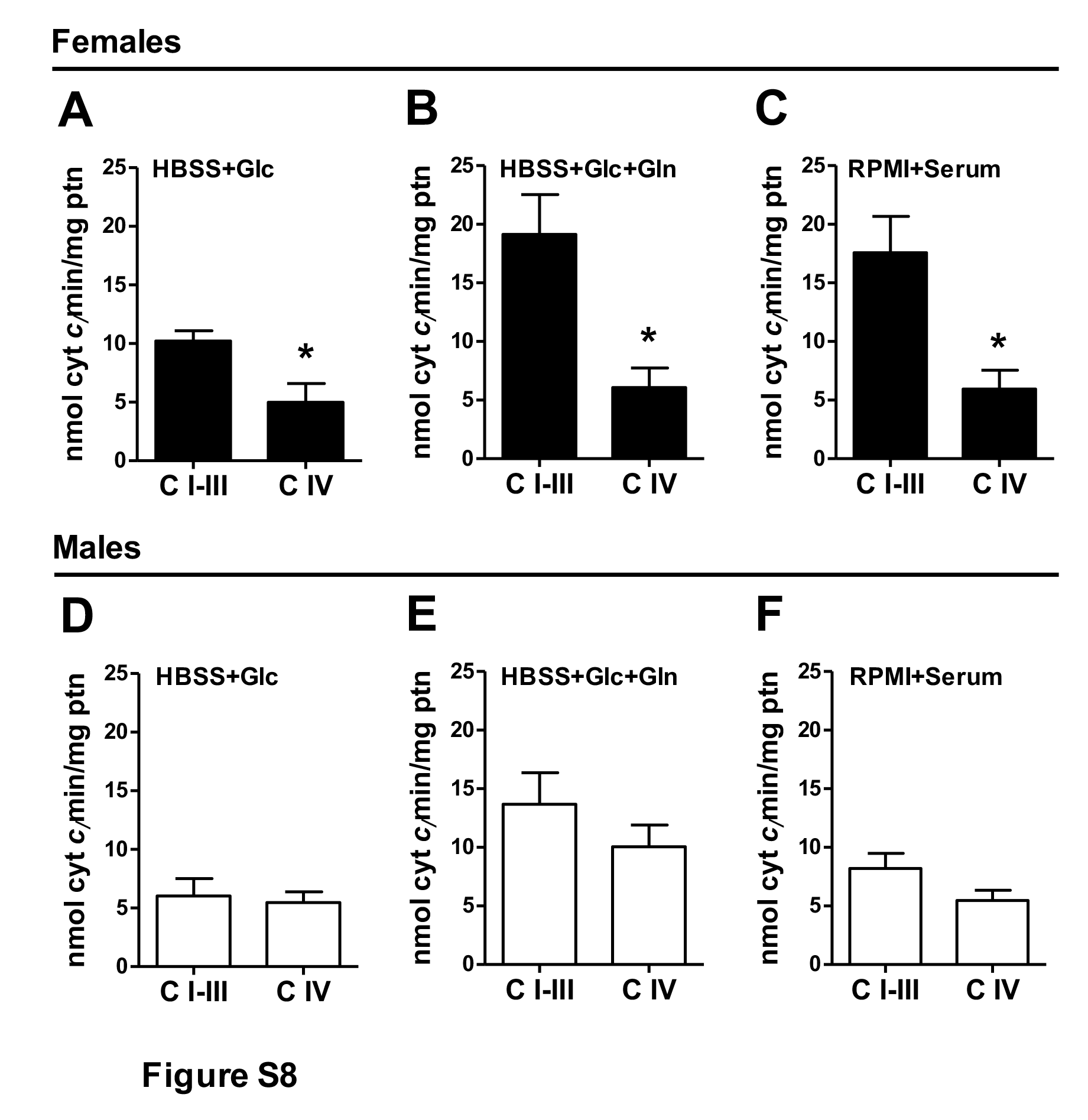

Supplement: S8 Fig — Complex I-III (C I-III) and complex IV (C IV) activities were measured in adult female (black bars) and male (white bars) worms kept in three different nutrient compositions, as following: HBSS + 5.5 mM glucose, HBSS + 5.5 mM glucose + 5.5 mM glutamine, or RPMI 1640 + serum. Data shown are mean ± SEM of at least five different experiments. Comparisons between groups were done by Student´s t-test (A) or Mann-Whitney´s test (B and C). Fig (A): * p<0.01 relative to C I-III. Fig (B): * p<0.02 relative to C I-III. Fig (C): * p<0.01 relative to C I-III. (TIF) [file pone.0158429.s008.tif]

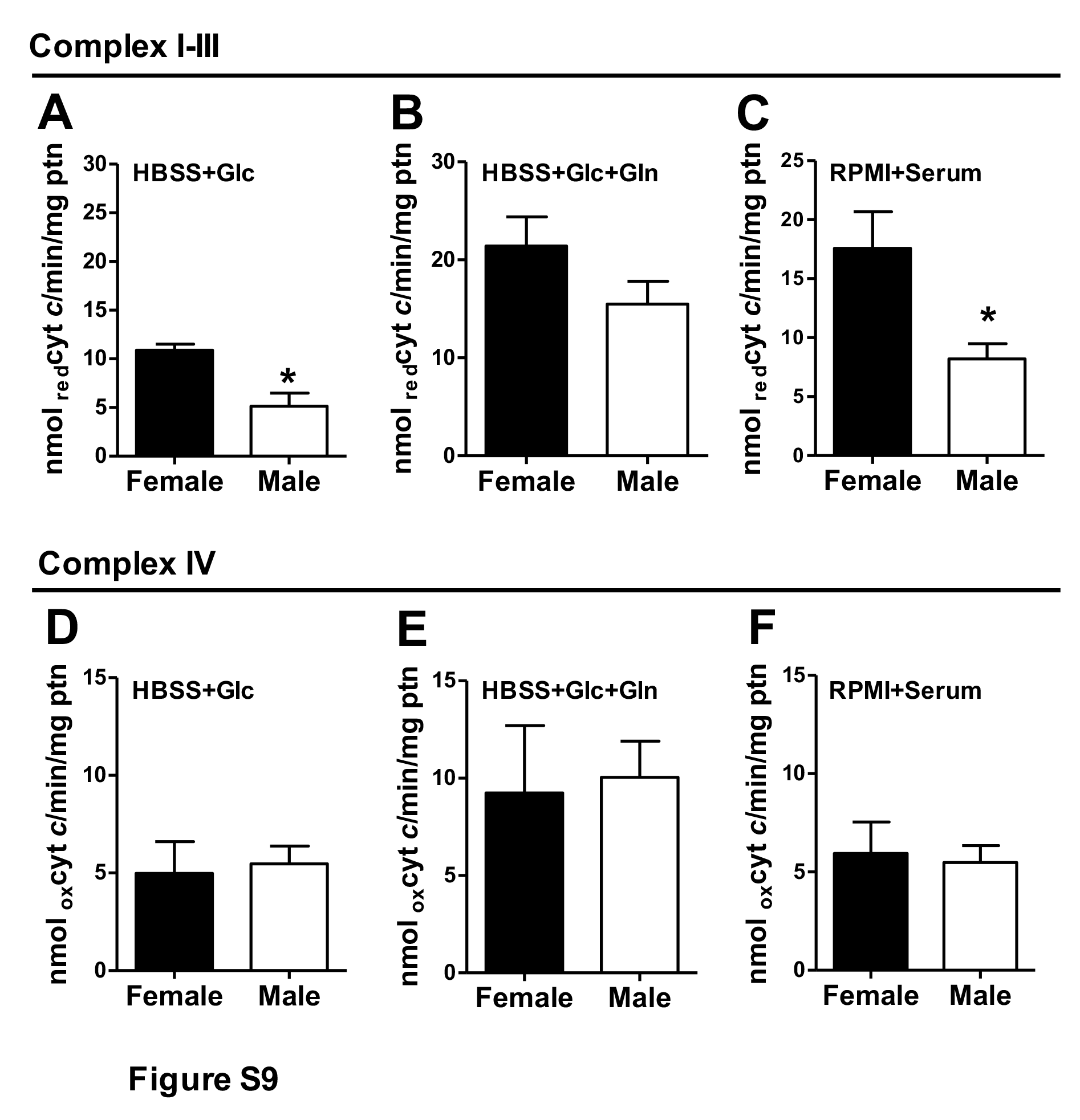

Supplement: S9 Fig — Complex I-III (C I-III) and complex IV (C IV) activities were measured in adult female (black bars) and male (white bars) worms kept in three different nutrient compositions, as following: HBSS + 5.5 mM glucose, HBSS + 5.5 mM glucose + 5.5 mM glutamine, or RPMI 1640 + serum. Data shown are mean ± SEM of at least five different experiments. Comparisons between groups were done by Student´s t-test (A) or Mann-Whitney´s test (B and C). Fig (A): * p<0.001 relative to females. Fig (C): * p<0.02 relative to females. (TIF) [file pone.0158429.s009.tif]

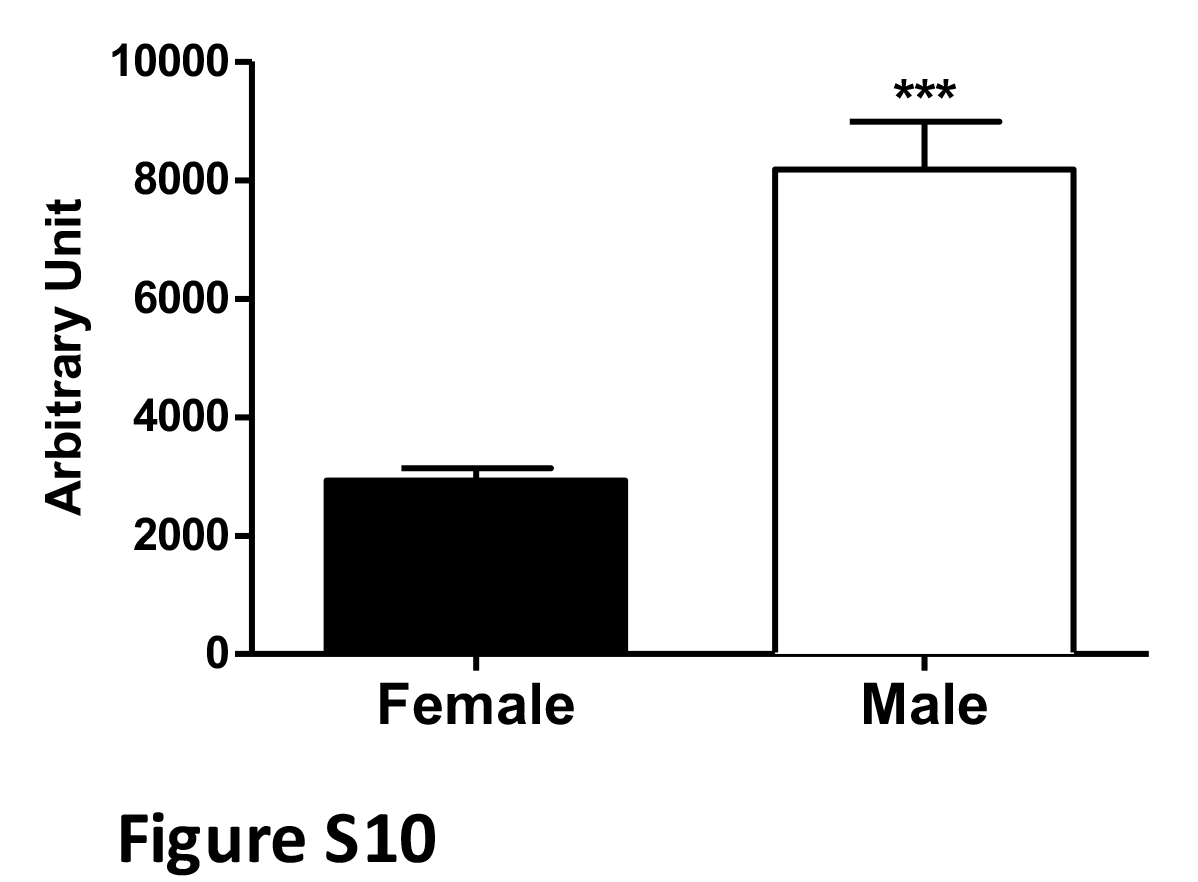

Supplement: S10 Fig — Worms were cultured for 24 h in RPMI + serum, and subsequently incubated in RPMI 1640 without phenol red supplemented with 1 μg/mL DAPI for 20 minutes at 37°C and 5% CO2 (See S1 Methods). Fluorescence microscopy images were collected for whole individual worms, the average fluorescence intensity was quantified and expressed as arbitrary units. Data are expressed as mean ± SEM of at least three different experiments. Comparisons between groups were done by using Mann-Whitney´s test. ***p<0.001. (TIF) [file pone.0158429.s010.tif]
