# Supplementary material for: Sexual Preferences in Nutrient Utilization Regulate Oxygen Consumption and Reactive Oxygen Species Generation in Schistosoma mansoni: Potential Implications for Parasite Redox Biology
Source: PLoS One. 2016 Jul 5;11(7):e0158429. doi: 10.1371/journal.pone.0158429 (PMC4933344; doi:10.1371/journal.pone.0158429)
Supplement: S1 Methods — (DOCX) [file pone.0158429.s011.docx]

**Supporting information methods:**

**Mitochondrial isolation**

Worms recovered from pre-incubation, as described in "*parasites*" section, followed a previously established method [45]. About 150 worms were gently homogenized in a Potter-Elvehjem tissue grinder using a glass pestle with 1mL of ice-cold isolation buffer (0.25 M sucrose;10 mM KH_2_PO_4_; 5 mM MgCl_2_; 20mM Tris; 1 mM EGTA; 0.5 % Bovine Serum Albumin (BSA), pH 7.2). The preparation was maintained at 4°C throughout the subsequent washing and centrifugation procedures. The sample was centrifuged at 300 x *g* for 5 min, the supernatant was collected and further centrifuged at 10,000 x *g* for 10 min. Then, the brown pellet was carefully re-suspended in approximately 0.1 mL of isolation buffer.

**Respirometry analyses on isolated mitochondria**

Respiration using isolated mitochondria was analyzed in a two-channel titration injection respirometer (Oxygraph-2k, Oroboros Instruments, Innsbruck, Austria) at 37°C. Aliquots corresponding about 250 μg of protein from freshly isolated mitochondria were transferred to the respirometer chambers. Respiration containing the "isolation buffer" in a final volume of 2.2 mL and allowed to equilibrate for about 15 minutes, with continuous stirring set up at 750 rpm. Then, the oxygen concentrations and the rates of oxygen consumption were simultaneously recorded in real time in both respirometer chambers by using the DatLab 5.0 software (Oroboros Inc., Austria). OCR was stimulated by addition of substrates to final concentrations of 10 mM glutamate + 1 mM malate (Glu-Mal) and 10 mM succinate (Suc). ATP synthesis coupled to oxygen consumption through the oxidative phosphorylation (OXPHOS) was promoted by the addition of 1 mM ADP and finally inhibited by complex III inhibitor antimycin a. The respiratory state 2 was determined by OCR stimulated with substrates and state 3 by OCR stimulated by addition of ADP. The OCR was normalized by protein mass determined from mitochondrial isolated fraction. The amount of protein obtained from mitochondrial fraction and injected in respirometer chamber were around 250 μg.

**DAPI staining**

Worms recovered from pre-incubation in RPMI 1640 without serum, as described in "*parasites*" section, were cultured in RPMI + Serum supplemented by 1% Streptomycin + Penicillin for 24 h at 37 ºC and 5% CO_2_. After that, worms were transferred separately to 96 wells plates and then incubated with DAPI 1 µg/mL for 30 minutes in RPMI 1640 without serum and phenol red, at a density of 1 worm/well, in darkness for 20 minutes at 37ºC and 5% CO_2_. Parasites were subsequently washed twice with 1x phosphate buffered saline (PBS) pH 7.4, re-suspended in 200 µL of 1x PBS and whole body fluorescence images were captured by epifluorescence microscopy using excitation at 358 nm and emission at 461 nm. Images were analyzed by AxioVision LE (Zeiss, USA) software. Average fluorescence intensity of masked images were registered and expressed as arbitrary fluorescence units.
